# Selenium Nanoparticles Synergize with a KRAS Nanovaccine against Breast Cancer

**DOI:** 10.1002/adhm.202401523

**Published:** 2024-08-29

**Authors:** Cláudio Ferro, Ana I. Matos, Luigia Serpico, Flavia Fontana, Jacopo Chiaro, Carmine D'Amico, Alexandra Correia, Risto Koivula, Marianna Kemell, Maria Manuela Gaspar, Rita C. Acúrcio, Vincenzo Cerullo, Hélder A. Santos, Helena F. Florindo

**Affiliations:** ^1^ Research Institute for Medicines iMed.Ulisboa Faculty of Pharmacy Universidade de Lisboa Lisbon 1649‐003 Portugal; ^2^ Drug Research Program Division of Pharmaceutical Chemistry and Technology Faculty of Pharmacy University of Helsinki Helsinki FI‐00014 Finland; ^3^ Department of Biomaterials and Biomedical Technology University Medical Center Groningen University of Groningen Ant. Deusinglaan 1 Groningen 9713 AV The Netherlands; ^4^ Drug Research Program Division of Pharmaceutical Biosciences Faculty of Pharmacy University of Helsinki Helsinki FI‐00014 Finland; ^5^ Department of Chemistry University of Helsinki Helsinki FI‐00014 Finland; ^6^ Helsinki Institute of Life Science (HiLIFE) University of Helsinki Helsinki FI‐00014 Finland

**Keywords:** breast cancer, immunotherapy, nanoparticles, nanovaccines, selenium

## Abstract

Selenium (Se) is an element crucial for human health, known for its anticancer properties. Although selenium nanoparticles (SeNPs) have shown lower toxicity and higher biocompatibility than other Se compounds, bare SeNPs are unstable in aqueous solutions. In this study, several materials, including bovine serum albumin (BSA), chitosan, polymethyl vinyl ether‐alt‐maleic anhydride, and tocopherol polyethylene glycol succinate, are explored to develop stable SeNPs and further evaluate their potential as candidates for cancer treatment. All optimized SeNP are spherical, <100 nm, and with a narrow size distribution. BSA‐stabilized SeNPs produced under acidic conditions present the highest stability in medium, plasma, and at physiological pH, maintaining their size ≈50–60 nm for an extended period. SeNPs demonstrate enhanced toxicity in cancer cell lines while sparing primary human dermal fibroblasts, underscoring their potential as effective anticancer agents. Moreover, the combination of BSA‐SeNPs with a nanovaccine results in a strong tumor growth reduction in an EO771 breast cancer mouse model, demonstrating a three‐fold decrease in tumor size. This synergistic anticancer effect not only highlights the role of SeNPs as effective anticancer agents but also offers valuable insights for developing innovative combinatorial approaches using SeNPs to improve the outcomes of cancer immunotherapy.

## Graphical abstract

Biocompatible SeNPs exhibit in vitro anticancer properties against both murine and human cancer cell lines. When combined with a KRAS_wt_ nanovaccine, they synergize in controlling tumor growth in EO771‐bearing mice. This synergy is evidenced by an increase in tumor‐infiltrating NK, B, and CD8^+^ T cells, as well as systemic activation of CD8^+^ T cells. In addition, SeNPs downregulate immunosuppressive Treg cells, highlighting their potential for cancer immunotherapy.

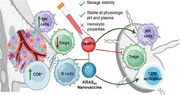

## 1 Introduction

Breast cancer is currently the second most frequently diagnosed cancer globally, accounting for 1 in 8 cancer diagnoses and ≈2.3 million new cases in both genders registered in 2022.^[^ ^1^ ^]^ Breast cancer represents a quarter of all female cancer cases and caused ≈685,000 deaths among women in 2022.^[^ ^2^ ^]^ It is a heterogeneous disease, including different subtypes. Among these, triple‐negative breast cancer (TNBC) is the most aggressive form, characterized by the absence of expression of the estrogen receptor (ER), progesterone receptor (PR), and human epidermal growth factor receptor 2 (HER2). TNBC usually manifests in younger patients and is associated with a poorer prognosis and higher mortality rate, with an average survival of 12–18 months.^[^ ^3^ ^]^ In addition, luminal B breast cancer is the most common subtype, accounting for 40% of all breast cancers. Studies indicate that patients with luminal B breast cancer have a higher local recurrence rate compared to patients with non‐luminal breast cancer.^[^ ^4^ ^]^ This subtype is characterized by being ERα negative, ERβ positive, PR positive, and HER2 positive.^[^ ^5^ ^]^

Conventional breast cancer treatments include chemotherapy and radiotherapy, which may have side effects and toxicity, as well as potential drug resistance. Moreover, these treatments cannot prevent possible late‐stage metastases, such as those found in the liver, lungs, and brain.^[^ ^3^, ^6^ ^]^ Furthermore, luminal B breast cancers are typically characterized by elevated levels of tumor‐infiltrating lymphocytes and immune checkpoints, making them viable targets for breast cancer immunotherapy.^[^ ^3^ ^]^ This approach has demonstrated efficacy in overcoming chemotherapy drug resistance and mitigating its side effects, resulting in a more favorable treatment response compared to monotherapy with either agent.^[^ ^7^ ^]^

To achieve more effective therapeutic options, nanotechnology‐based platforms have increasingly leveraged particles' passive or active targeting of tumors. Nanosystems preferentially accumulate in the tumor microenvironment (TME) through passive targeting, facilitated by the enhanced permeability and retention (EPR) effect induced by the abnormal proliferation of endothelial cells. This results in a permeable and complex tumor vasculature that enables the extravasation of nanoparticles (NPs) into the TME.^[^ ^8^ ^]^ To maximize their accumulation at the tumor site and evade clearance by the reticuloendothelial system and kidneys, NPs should ideally fall within a size range of 10–200 nm.^[^ ^9^ ^]^ Additionally, certain pH‐responsive NPs are designed to degrade specifically in the acidic environment of cancer cells.^[^ ^10^, ^11^ ^]^ Active targeting of NPs has also been implemented, achieved by decorating these carriers with ligands that have high‐affinity for cancer cell markers, such as aptamers and antibodies.^[^ ^8^ ^]^

Among nanosystems, selenium nanoparticles (SeNPs) have demonstrated potent antiproliferative properties against several types of cancers, including breast and lung cancers.^[^ ^12^, ^13^ ^]^ Selenium (Se) is incorporated into several selenoproteins, which are known for their roles in protecting against reactive oxygen species (ROS) and modulating immune and inflammation processes.^[^ ^14^, ^15^ ^]^ Consequently, Se has garnered considerable attention for its potential to prevent or treat several diseases, including cancer, as malignant cells are more susceptible to Se‐induced cytotoxic effects. However, Se‐containing compounds have exhibited toxicity against healthy cells, limiting their therapeutic application as antitumor agents due to a narrow therapeutic window.^[^ ^14^, ^16^ ^]^ In contrast, SeNPs have emerged as a promising alternative, demonstrating lower toxicity and enhanced biocompatibility compared to organic and inorganic Se compounds.^[^ ^14^, ^17^ ^]^ Furthermore, SeNPs present superior antitumor properties by selectively inducing ROS overexpression in cancer cells, leading to mitochondrial dysregulation and apoptosis.^[^ ^14^, ^18^ ^][^ ^16^ ^]^ In addition, SeNPs have shown promise in cancer immunotherapy by downregulating cancer human leukocyte antigen‐E expression, thereby increasing cancer cell sensitivity to natural killer (NK) cells. Moreover, SeNPs promote T‐cell activation and macrophage polarization toward an M1‐like macrophage phenotype.^[^ ^19^ ^]^

To date, several methods have been used to produce SeNPs, including biosynthesis, chemical reduction, and physical synthesis using gamma radiation to reduce Se ions.^[^ ^16^ ^]^ Bare SeNPs are unstable in aqueous suspensions, precipitating as gray/black Se^[^ ^20^, ^21^ ^]^ thereby requiring stabilizers to preserve their size and bioactivity.^[^ ^21^, ^22^ ^]^ Several compounds have been identified as stabilizers for SeNPs, including chitosan,^[^ ^23^ ^]^ bovine serum albumin (BSA),^[^ ^24^ ^]^ and several polymers.^[^ ^25^ ^]^ However, inconsistencies arise as each study employs different conditions for SeNP production, even when using the same methodology and stabilizer.^[^ ^16^, ^26^, ^27^ ^]^ Thus, establishing optimized SeNP production procedures is crucial for understanding how they influence SeNP properties, such as size, polydispersity index (PdI), and ζ‐potential. In addition, it is essential to assess the impact of stabilizers on the biochemical properties of SeNPs.

Here, SeNPs were synthesized by chemical reduction using four different materials as stabilizers: a polysaccharide, chitosan; a protein, BSA; and two polymers poly(methylvinylether‐co‐maleic anhydride) (PMVE‐MA) and tocopherol polyethylene glycol succinate (TPGS). BSA has been previously employed for the production of biocompatible SeNPs,^[^ ^28^, ^29^ ^]^ demonstrating antibacterial^[^ ^24^ ^]^ and anticancer properties.^[^ ^29^ ^]^ Chitosan has also been extensively investigated for SeNP stabilization,^[^ ^16^ ^]^ particularly in cancer therapy applications.^[^ ^30^, ^31^ ^]^ PMVE‐MA has attractive properties such as biocompatibility, safety, and bio‐adhesivity,^[^ ^32^ ^]^ and has been used in the development of microneedles,^[^ ^33^ ^]^ chemotherapeutic nanocarriers,^[^ ^32^, ^34^ ^]^ and, more recently, in the formation of nanocapsules containing a core of Selol, an organic selenium donor.^[^ ^35^ ^]^ Additionally, TPGS, a water‐soluble derivative of vitamin E, has been documented for its role in producing nanosystems delivering anticancer agents.^[^ ^36^, ^37^ ^]^ The production of SeNPs was optimized for each stabilizer, addressing parameters such as sonication requirements, reaction temperature, stirring speed, stabilizer concentration, and dialysis temperature.

After optimizing the production of SeNPs, our study elucidated the profound influence of stabilizers on various properties of SeNPs, including their storage and stability profiles, as well as their anticancer efficacy against both human and murine cell lines. Specifically, our findings reveal that SeNPs stabilized with BSA presented superior stability at physiological pH and in plasma while demonstrating potent antitumor activity in both murine and human cancer cell lines.

Furthermore, we evaluated the antitumor efficacy of the optimized BSA‐SeNPs in vivo using the EO771 breast cancer mouse model. Our results demonstrate that EO771‐bearing mice treated with SeNPs in combination with a therapeutic nanovaccine, which co‐delivered breast cancer‐associated antigens and immune potentiators, effectively controlled tumor growth and modulated both systemic and tumor‐infiltrating immune cell populations.

## 2 Results and Discussion

### 2.1 Synthesis of Selenium Nanoparticles (SeNPs)

This study focused on the production of stable SeNPs with anticancer properties and further investigated the parameters that most affect the physicochemical properties and stability of SeNPs. To this end, we explored two methodologies for producing SeNPs. The impact of different stabilizers and synthetic parameters on NP size, PdI, and ζ‐potential was also evaluated. Overall, the targeted specifications for SeNP included an average mean diameter between 10 – 100 nm and a narrow PdI (< 0.2) to exploit the EPR effect while avoiding kidney clearance.^[^ ^9^, ^38^ ^]^ According to the literature, a higher absolute ζ‐potential is considered a contributing factor to the long‐term stability of NPs, effectively preventing aggregation.^[^ ^39^ ^]^ Therefore, the ζ‐potential of the prepared SeNPs was systematically measured to identify the most stable formulations.

**Figure 1:**
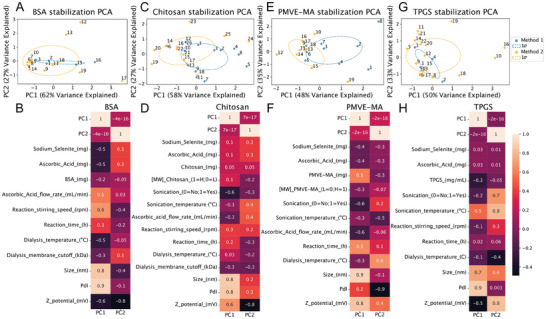
Principal components analysis (PCA) of the production procedure and conditions used to synthesize selenium nanoparticles (SeNPs), in which each number corresponds to data points. A,C,E,G) Blue corresponds to method 1 and orange to method 2. B,D,F,H) Heatmap displaying the corresponding Pearson's Correlations coefficients of the principal components (PC) and the different production parameters of the NPs’ datasets. Data was analyzed using Python (Anaconda Environment).

Accordingly, the following parameters were explored to understand their impact on SeNP properties: the concentration of reagents, the need for sonication and the temperature during sonication, the flow rate of ascorbic acid addition, stirring speed, reaction time, dialysis temperature and membrane pore size. Studies have indicated that sodium selenite and ascorbic acid react in a molar ratio of 1:2; however, an excess of ascorbic acid provides a more favorable reduction environment for the production of SeNPs and helps prevent their potential oxidation.^[^ ^24^, ^40^ ^]^ To assess the differences among the particles produced during the optimization process, we employed Principal Component Analysis (PCA), a methodology often used for dimensionality reduction problems, to analyze their physical properties, such as size, PdI, and ζ‐potential. PCA revealed the two principal components (PC) responsible for most of the dataset variance, resulting in a 2‐dimensional dataset representation. Ellipses were traced for each PCA to better display the groups of data points corresponding to particles produced with methods 1 and 2, representing one standard deviation of the distribution (**Figure** **1**).

Regarding BSA‐SeNPs, the first two PCs accounted for 62% and 27% of the total dataset variance (Figure 1A). The overlap between the sets of particles produced using the two methods indicated that these BSA‐SeNPs presented similar characteristics. However, fewer data points were available for BSA‐SeNPs prepared using method 1, as these particles presented larger diameters and lower positive ζ‐potential compared to those obtained using method 2 (datapoints numbers 1 and 9 in Supplementary Excel File – PCA dataset). Among the physical properties analyzed, size and PdI were the variables that contributed most to the PC1 and PC2, which might be attributed to the differences in reaction stirring speed (Figure 1B). Specifically, we observed that method 2 produced relatively homogenous small BSA1‐SeNPs, except for a few outliers that could be explained by other influencing parameters. In contrast, method 1 yielded a broader size range, as indicated by the corresponding peak (Figure S1A, Supporting Information). Furthermore, the reaction stirring speed appeared to play a crucial role in determining BSA‐SeNP size, while the pH of the reaction mixture influenced both the PdI and the ζ‐potential (Figure S2A, Supporting Information). Adjusting the pH to 1 (BSA1‐SeNPs) increased the positive charge of the SeNPs’ surface, while alkalinization of the reaction mixture to pH 12 (BSA12‐SeNPs) reverted the SeNPs’ charge to negative values.

As for the SeNPs stabilized with chitosan (Chitosan‐SeNPs), the first two PCs accounted for 60% and 27% of the total variance. We observed only a partial overlap between the two sets of particles, leading us to conclude that the two methods produce different particles only under certain conditions (Figure 1C). For this type of SeNPs, ζ‐potential was the physical property that most significantly contributed to the PC. Additionally, we noted a weak correlation between the PC2 and ascorbic acid flow rate or size (Figure 1D). However, both method 1 and method 2 yielded similar Chitosan‐SeNPs with closely distributed size, PdI, or ζ‐potential values (Figure S1B, Supporting Information). Moreover, the use of sonication was the parameter that primarily affected the size and PdI (Figure S2B, Supporting Information).

For SeNPs stabilized with PMVEMA (PMVEMA‐SeNPs), the first two PCs accounted for 53% and 27% of the dataset variance. The overlap between the two sets of particles suggested that the properties of the SeNPs were not highly affected by the production method used (Figure 1E). Once again, size and PdI appeared to be the variables that contributed most to PC1 and PC2, possibly depending on several factors such as the amount of polymer, the use of sonication, and the dialysis temperature (Figure 1F). Accordingly, differences in size and ζ‐potential were observed between particles produced by the two methods. Additionally, similar to the observations with BSA‐SeNPs, method 2 yielded more homogeneous distributions and narrower peaks for all analyzed features (size, PdI, and ζ‐potential), whereas wider peaks were obtained using method 1. (Figure S1C, Supporting Information). Furthermore, the size of PMVEMA‐SeNPs was predominantly influenced by the amounts of sodium selenite and ascorbic acid, the use of sonication, and the flow rate of ascorbic acid addition. Following the optimization of PMVEMA‐SeNPs, the flow rate was standardized to 8 mL min^−1^ for all SeNPs, regardless of the stabilizers used. The dialysis temperature affected the PdI of PMVEMA‐SeNPs, while their ζ‐potential was not influenced by any condition (Figure S2C, Supporting Information).

Lastly, to produce SeNPs stabilized with TPGS (TPGS‐SeNPs), the first two PCs accounted for 49% and 33% of the dataset variance. We observed nearly complete overlap between the two sets of particles produced with the two methods, suggesting that both production methods led to similar TPGS‐SeNPs (Figure 1G). Nevertheless, the use of sonication, as well as the PdI and the ζ‐potential, appeared to be the variables that predominantly influenced PC1 and PC2 (Figure 1H). Moreover, the similar distributions of properties observed in TPGS‐SeNPs prepared using method 1 or method 2 indicated that the system was not significantly affected by the method of preparation. Considering these results, and since method 1 required considerably higher amounts of the surfactant, no further formulations were prepared, which justifies the limited number of data points available for TPGS‐SeNPs prepared using method 1 (Figure S1D, Supporting Information). Nonetheless, it was evident that the size and PdI of TPGS‐SeNPs were negatively affected by the sonication temperature (Figure S2D, Supporting Information).

Overall, the data obtained from this analysis allowed us to define the optimal parameters for the synthesis. The selected procedure, method 2, included a flow rate of ascorbic acid addition of 8 mL minute^−1^, a reaction time of 30 minutes, stirring at 400 rpm, and the use of a dialysis membrane with a molecular weight cut‐off of 12–14 KDa for all the stabilizers used. Subsequently, these production conditions were applied to prepare BSA‐SeNPs, Chitosan‐SeNPs, PMVEMA‐SeNPs, and TPGS‐SeNPs.

For BSA‐SeNPs, 8 mL of an aqueous ascorbic acid solution (50 mM) was added to 1 mL of a mixture containing BSA (10 mg mL^−1^) and sodium selenite (100 mm). Following mixing, the pH of the reaction medium was adjusted to 1 or 12 to produce BSA1‐SeNPs and BSA12‐SeNPs, respectively. The BSA‐SeNP suspension was then dialyzed at 4°C.

To produce Chitosan‐SeNPs, 1 mL of an aqueous solution of chitosan (5 mg mL^−1^) dissolved in 4% (v/v) acetic acid and sodium selenite (50 mM) were mixed under sonication at 4 °C. Subsequently, 8 mL of an aqueous ascorbic acid solution (25 mM) was added. After the reaction, the Chitosan‐SeNPs suspension was dialyzed at room temperature.

PMVEMA‐SeNPs were produced by sonicating 1 mL of an aqueous solution of PMVE‐MA (1 mg mL^−1^) and sodium selenite (100 mm) at room temperature. Then, 8 mL of an aqueous ascorbic acid solution (50 mm) was added, followed by dialysis at room temperature.

Finally, for TPGS‐SeNPs, 8 mL of an aqueous ascorbic acid solution (50 mm) was added to 1 mL of a mixture of TPGS (1 mg mL^−1^) and sodium selenite (100 mm). The resulting TPGS‐SeNPs were dialyzed at room temperature.

### 2.2 Physicochemical Properties of SeNPs as a Function of the Stabilizer

**Figure 2:**
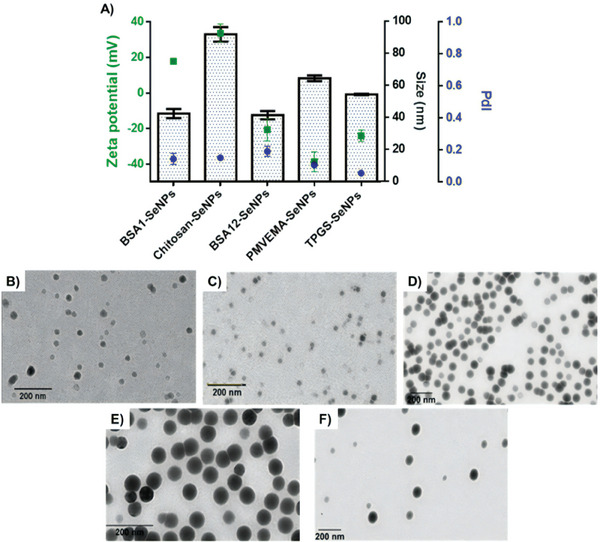
Characterization of the SeNPs produced using the selected production method 2 and the reaction conditions identified as optimal: A) size (nm) (in columns), PdI (blue), and ζ‐potential (mV) (green). TEM images of B) BSA1‐SeNPs, C) BSA12‐SeNPs, D) Chitosan‐SeNPs, E) PMVEMA‐SeNPs, and F) TPGS‐SeNPs. Scale bar: 200 nm.

The SeNPs were synthesized by the chemical reduction of Se(IV) in sodium selenite to elemental Se, using ascorbic acid under optimized conditions for each stabilizer. Subsequently, the resulting SeNPs were characterized by Dynamic Light Scattering (DLS) and Transmission Electron Microscopy (TEM) techniques (**Figure** **2**). DLS measurements revealed that all SeNPs presented average sizes below 100 nm. Specifically, the average sizes were 91.78 ± 4.53 for Chitosan‐SeNPs, 64.33 ± 1.79 for PMVEMA‐SeNPs, and 54.21 ± 0.43 for TPGS‐SeNPs. BSA‐SeNPs were the smallest, with sizes below 50 nm, measuring 42.30 ± 2.81 for BSA1‐SeNPs and 41.28 ± 2.59 for BSA12‐SeNPs (Figure 2A). Therefore, it is anticipated that all SeNPs will efficiently extravasate passively from the permeable tumor vasculature into the TME.^[^ ^10^, ^11^ ^]^

All five types of SeNPs presented PdI values <0.20, indicating adequate monodispersity. The ζ‐potential values varied depending on the stabilizer and the pH conditions. BSA1‐SeNPs and Chitosan‐SeNPs were positively charged, likely due to the protonation of the ‐NH_3_ groups in the BSA and chitosan molecules, while the other SeNPs displayed negative ζ‐potential. These negative surface charges could be attributed to the alkalinization of the reaction mixture in BSA12‐SeNPs production or the presence of ester groups in TPGS and PMVE‐MA polymers (Figure 2A).

TEM images confirmed that all SeNPs presented a spherical, symmetric morphology, and uniform distribution (Figure 2B–F). In general, the TEM data corroborated the DLS measurements, although with some discrepancies. Specifically, BSA1‐SeNPs, BSA12‐SeNPs, Chitosan‐SeNPs, TPGS‐SeNPs and PMVEMA‐SeNPs displayed average sizes of 47.64 ± 6.84 nm (Figure 2B), 33.82 ± 6.26 nm (Figure 2C), 60.39 ± 10.41 nm (Figure 2D), 67.95 ± 19.16 nm (Figure 2E), and 60.79 ± 8.43 nm (Figure 2F), respectively. These variations were expected and can be explained by the broadening of size distributions, as DLS analysis in volume modality tends to highlight the largest particles within the entire sample.^[^ ^41^ ^]^

Additionally, the number of particles, the Se concentration, and the Se amount per particle were quantified (Table S1, Supporting Information). BSA1‐SeNPs presented higher Se concentration, particle concentration, and Se amount per particle compared to BSA12‐SeNPs, indicating that the pH of the reaction influenced both the formation of SeNPs and the Se content per particle. This observation aligns with the larger size of BSA1‐SeNPs measured by TEM. Overall, BSA1‐SeNPs presented the highest Se content, while TPGS‐SeNPs samples exhibited the largest number of particles. Moreover, PMVEMA‐SeNPs and BSA1‐SeNPs contained the highest Se quantity per particle, with 2.4 × 10^−13^ and 4.0 × 10^−13^ mg per particle, respectively.

**Figure 3:**
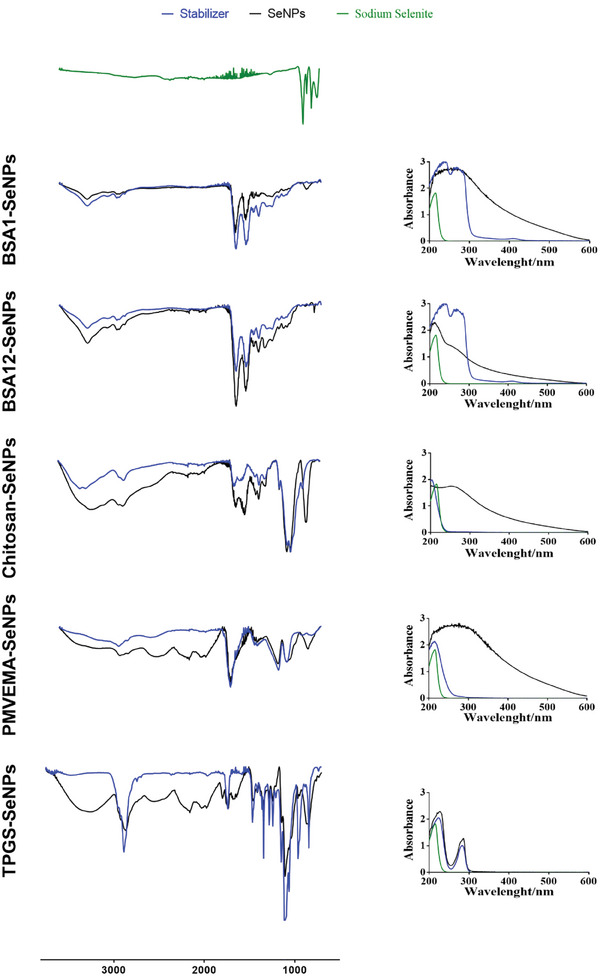
ATR‐FTIR and UV/Vis spectroscopy analysis of SeNPs produced using the optimized production method and reaction conditions (BSA1‐SeNPs; BSA12‐SeNPs; Chitosan‐SeNPs; PMVEMA‐SeNPs; TPGS‐SeNPs).

UV/Vis spectroscopy and Attenuated Total Reflectance Fourier transform IR spectroscopy (ATR‐FTIR) analysis were performed to confirm the presence of Se and the stabilizer in each SeNP (**Figure** **3**). In general, SeNPs presented a maximum absorbance of ≈260 nm.^[^ ^17^, ^42^, ^43^ ^]^ In addition, Energy‐Dispersive X‐ray Spectroscopy (EDS) analysis was performed to confirm the presence of Se (Figure S3, Supporting Information), as several stabilizers exhibited similar absorbance peaks. Compared to the background with no SeNPs (in red), all SeNPs showed X‐ray lines of Se, confirming its presence.

Furthermore, chemical characterization was conducted to confirm the presence of stabilizers by comparing the ATR‐FTIR spectra of sodium selenite, SeNPs, and the respective stabilizer (Figure 3). The bands observed in the sodium selenite spectra were absent in the spectra of all SeNPs, indicating the transformation of the starting product during the reaction. Moreover, a comparison of the spectra of each SeNP with its corresponding stabilizer confirmed the presence of the stabilizers within the SeNP structure. In BSA1‐SeNPs and BSA12‐SeNPs, characteristic bands of pure BSA were detected, including peaks at 3280 cm^−1^ (N‐H stretching), 2934 cm^−1^ (N‐H stretching of NH_3_^+^ free ion), strong characteristic bands at 1640 cm^−1^ (C═O of amide), 1535 cm^−1^ (C–N stretching and N–H bending vibrations), 1393 cm^−1^ (CH_2_ bending groups) and 1260 cm^−1^ (C–N stretching and N–H bending).^[^ ^44^ ^]^ Therefore, this analysis confirmed the effective presence of BSA in the SeNPs at both investigated pH values.

Chitosan‐SeNPs presented typical bands of pure chitosan, including peaks at 2920 and 2880 cm^−1^ (C─H stretching), 1626 cm^−1^ (C═O stretching associated with the primary amide), 1564 cm^−1^ (N‐H bending of the ‐NH_2_ groups), 1377 cm^−1^ (C─H bending), and a strong band at 1065 cm^−1^ (CO‐O‐CO stretching, due to the bridge O between the glucosamine residues).^[^ ^45^ ^]^ Additionally, a strong broadband close to 850 cm^−1^ was observed only in the Chitosan‐SeNPs spectrum. While chitosan presented two bands ≈3250 cm^−1^ (related to the overlapping of the ‐OH and ‐NH stretching, as well as intramolecular hydrogen bonds),^[^ ^45^, ^46^ ^]^ the Chitosan‐SeNPs spectrum included only one of these bands. Therefore, we hypothesize that a new interaction might have occurred during SeNP production due to the reaction environment.

PMVEMA‐SeNPs presented all bands typically observed for PMVE‐MA, including a strong band close to 1700 cm^−1^ corresponding to the C═O stretching due to the carbonyl groups, as well as, bands at 1402 cm^−1^ resulting from the C─H bending, and strong bands at 1185 and 1082 cm^−1^ representing the stretching vibrations of C─H and C─OH, respectively, attributed to the several functional groups in the PMVE‐MA structure. Additionally, a characteristic band absorbing at 2935 cm^−1^, likely due to the C─H stretching vibration of the O─CH─O, was observed.^[^ ^47^ ^]^ However, the ATR‐FTIR spectrum of PMVEMA‐SeNPs presented three strong peaks at 854, 1978, and 2160 cm^−1^, characteristic of ─C═C─, ─C═C═C─, and ─C═C═O bonds, respectively, suggesting the formation of new bonds.

On the other hand, the spectra of TPGS‐SeNPs presented similar bands to the TPGS spectrum, including bands at 2867 cm^−1^ representing the C─H stretching bonds, 1737 cm^−1^ representing the C═O bonds in the esters groups, and strong bands at 1105 and 1278 cm^−1^ representing the typical C─O─C stretching vibrations of the repeated ─OCH_2_CH_2_ units of TPGS and the ─COO bond stretching vibrations, respectively. However, the intensity of these last two bands in the TPGS‐SeNPs spectrum was weaker than the TPGS characteristic band at 3400 to 3650 cm^−1^,^[^ ^48^ ^]^ which was also wider, indicating that some carbonyl bonds might have been broken into alcohol groups, such as intermolecular bonded and/or free alcohols. Bands at 2162 and 2023 cm^−1^ were also observed, which might indicate that during the preparation of TPGS‐SeNPs, new bonds were formed.^[^ ^49^ ^]^

To further confirm the presence of the stabilizer in the SeNPs, elemental analysis was performed to assess the percentage of carbon (C), nitrogen (N), hydrogen (H), and sulfur (S) (Table S2, Supporting Information). The percentages of the elements in each type of SeNP were consistent with the respective stabilizers used, confirming the presence of the compounds used for SeNP stabilization in their structure. S was only relevant in the BSA‐SeNPs due to the cysteine residues in the BSA structure.^[^ ^50^ ^]^ Notably, SeNPs stabilized with BSA, and chitosan presented considerable amounts of nitrogen (N), consistent with the chemical formula of both stabilizers. However, while PMVEMA‐SeNPs and TPGS‐SeNPs mainly consisted of Se, the stabilizers were responsible for 10% of their composition. Moreover, some SeNPs, particularly BSA12‐SeNPs, contained higher percentages of the stabilizer, indicating varying amounts of stabilizers within each type of SeNPs are necessary to produce stable formulations.

### 2.3 SeNPs Stability and the Impact of Cryoprotectants during Freeze‐Drying

**Figure 4:**
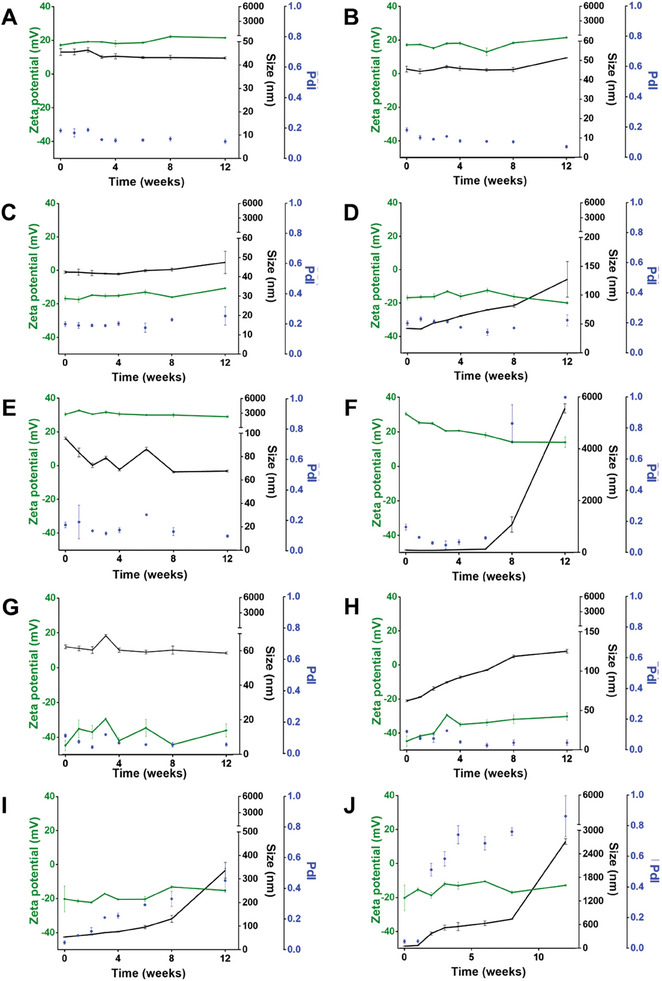
Storage stability of SeNPs: variations on size (black), PdI (blue), and ζ‐potential (grey) of the different SeNPs stored at A,C,E,G,I) 4 °C and B,D,F,H,J) room temperature for 12 weeks: A,B) BSA1‐SeNPs; C,D) BSA12‐SeNPs; E,F) Chitosan‐SeNPs; G,H) PMVEMA‐SeNPs; I,J) TPGS‐SeNPs.

The stability of the SeNPs was evaluated both at room temperature and at 4 °C, for 12 weeks by assessing their size and ζ potential (**Figure** **4**). All SeNPs presented higher stability when stored at 4 °C compared to room temperature, suggesting that low temperatures can delay material degradation, in line with existing literature on SeNPs and other nanomaterials.^[^ ^51^ ^]^

BSA1‐SeNPs (Figure 4A,B) maintained the PdI below 0.20 and the ζ potential above +15 mV throughout the 12 weeks, while sizes were consistently between 40 and 50 nm, except for week 12 when stored at room temperature. BSA12‐SeNPs (Figure 4C,D) showed greater instability, with size increasing over time, especially after 12 weeks of storage at room temperature, reaching values above 100 nm. In addition, the PdI became >0.2, and oscillation of ζ potential values was observed. Chitosan‐SeNPs tended to decrease in size over time, dropping below 70 nm when stored at a lower temperature, (Figure 4E), but exhibited significant size increases when stored at room temperature, exceeding 1000 nm at week 8 and 5000 nm at week 12. Furthermore, PdI values above 0.80 and decreased ζ potential were observed for Chitosan‐SeNPs, indicating continuous aggregation and sedimentation (Figure 4F).

PMVEMA‐SeNPs maintained stability when stored at 4 °C, with both size and ζ potential preserved over 12 weeks (Figure 4G). However, at room temperature, size increased over time, reaching 125 nm after 3 months, with minimal changes in PdI, although the ζ potential tended to be less negative (Figure 4H).

TPGS‐SeNPs demonstrated instability at 4 °C and room temperature (Figure 4I,J), with size and PdI already exceeding 350 nm and 0.50, respectively, at week 2. Both parameters continued to increase over time, suggesting aggregate formation. Even at 4 °C, TPGS‐SeNPs exhibited instability, with a final size and PdI of 335 nm and 0.45, respectively, indicating aggregate formation even at lower temperatures.

TEM analysis after 4 weeks of storage supported the hypothesis that SeNPs are more stable at 4 °C than at room temperature (Figure S4, Supporting Information). Overall, it is evident that BSA1‐SeNPs exhibited the highest stability, maintaining their size during long‐term storage at both 4 °C and room temperature, while TPGS‐SeNPs became unstable after 4 weeks, making them unsuitable for long‐term studies.

Lyophilization is commonly used to improve the stability of pharmaceutical formulations, including NPs, offering solid‐state administration routes.^[^ ^52^ ^]^ However, lyophilization can induce aggregation, fusion, or content leakage, as observed for SeNPs after the rehydration.^[^ ^53^ ^]^ Trehalose and sucrose have been studied as cryoprotectants during freeze‐drying, with low molecular weight sugars like trehalose leading to better stability than high molecular weight sugars such as maltodextrin. However, the concentration of the cryoprotectant is also crucial for stability during freeze‐drying.^[^ ^52^ ^]^ We evaluated the cryoprotectant potential of both trehalose and sucrose at concentrations of 5, 10, 20, and 40 mg mL^−1^, after rehydration (Figure S5, Supporting Information).

Our data indicated that BSA‐SeNPs were unaffected by the lyophilization, as size, PdI, and ζ‐potential after resuspension were similar to fresh BSA‐SeNPs (Figure S5A,B, Supporting Information). However, the size and PdI of Chitosan‐SeNPs, TPGS‐SeNPs, and PMVEMA‐SeNPs increased without a cryoprotectant (Figure S5C–E, Supporting Information). Interestingly, when cryoprotectants were used, PMVEMA‐SeNPs size and PdI were similar to fresh samples, while Chitosan‐SeNPs and TPGS‐SeNPs sizes remained above 100 nm, suggesting insufficient preservation of their characteristics. These results highlight the versatility and stability of BSA1‐SeNPs after lyophilization compared to PMVEMA‐SeNPs, which require a cryoprotectant, as well as Chitosan‐SeNPs and TPGS‐SeNPs, which remained unstable even when cryoprotectants were used. Furthermore, no significant difference was observed between trehalose and sucrose, indicating similar protective effects during the lyophilization.

### 2.4 Stability of SeNPs in Physiological Medium, Plasma, and Across Various pH Conditions

Once in contact with serum or plasma, the properties of NPs can be affected by several macromolecules, primarily serum proteins. These proteins typically bind to NP surfaces via non‐covalent forces such as hydrogen bonding, Van der Walls forces, as well as hydrophobic and electrostatic interactions.^[^ ^54^ ^]^ Therefore, proteins that rapidly exchange binding on the NP surface create an outer protein layer known as a “soft corona”, while a “hard corona” forms as an inner layer of strongly bound proteins, potentially changing NP properties within the bloodstream.^[^ ^54^ ^]^ Understanding the composition of the “hard corona” is important for defining the systemic properties of NPs, which in turn affect biodistribution, accumulation, interactions with the cell membranes, and therapeutic efficacy.^[^ ^54^ ^]^ The binding of serum proteins, including immunoglobulins and fibrinogen, can increase recognition and clearance by the mononuclear phagocytic system.^[^ ^54^ ^]^ However, the formation of the protein coronas around NPs varies based on factors such as size, shape, and charge.^[^ ^54^ ^]^ For example, positively charged NPs are more prone to protein adsorption, aggregation, and opsonization, leading to a shorter circulation half‐life.^[^ ^55^, ^56^ ^]^ In addition, NP aggregation can occur due to factors like high ionic strength, salt content, and biomolecules acting as bridging agents among NPs, resulting in large clusters with altered diffusion and stability profiles, potentially impacting cellular uptake and toxicity.^[^ ^56^ ^]^

**Figure 5:**
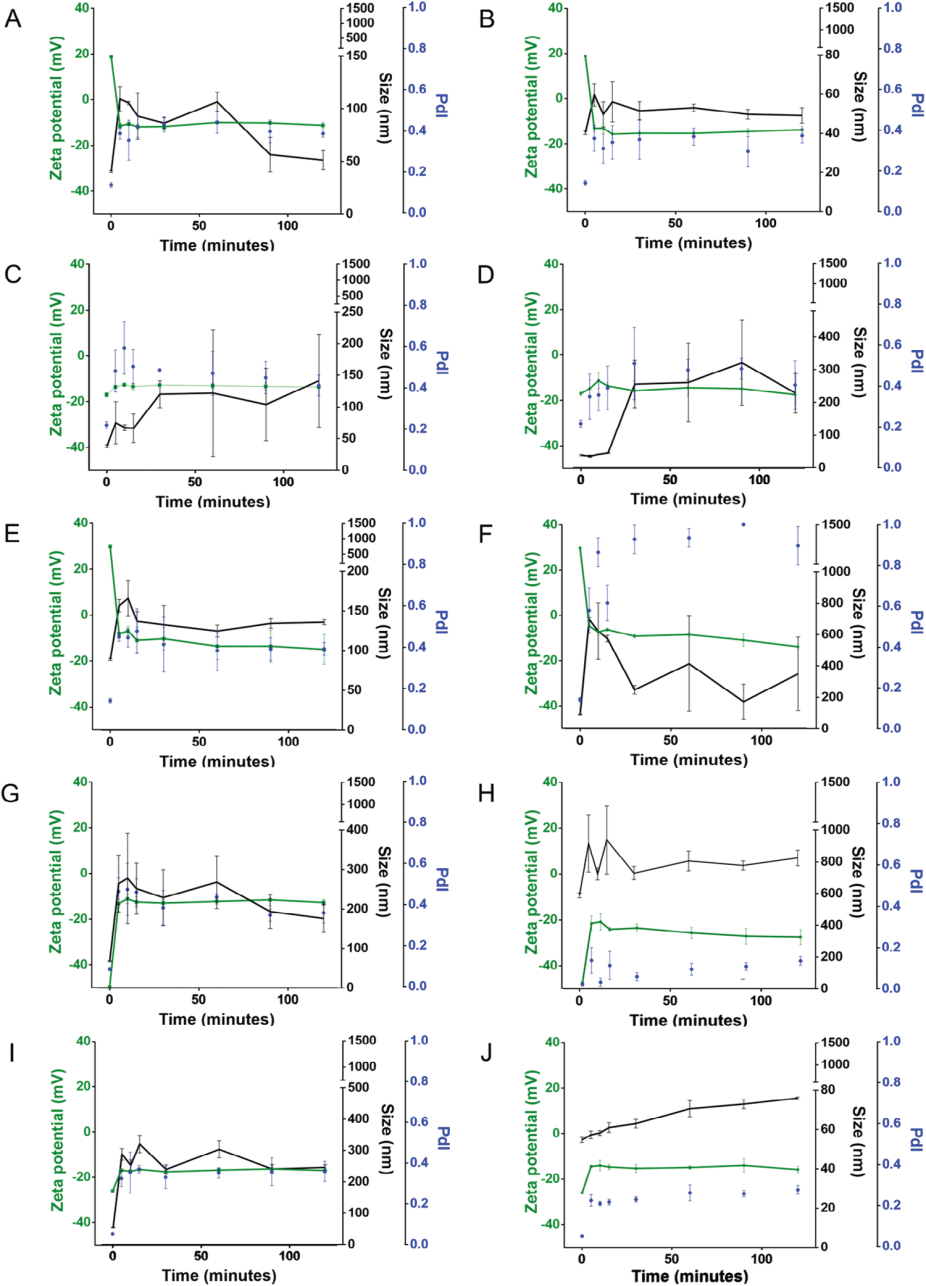
A,C,E,G,I) Stability profiles of SeNPs in human plasma and B,D,F,H,J) RPMI cell medium supplemented with 10% of FBS at 37 °C for 2 hours: A,B) BSA1‐SeNPs; C,D) BSA12‐SeNPs; E,F) Chitosan‐SeNPs; G,H) PMVEMA‐SeNPs; I,J) TPGS‐SeNPs – analysis of SeNP size (black), PdI (blue) and ζ‐potential (green). Error bars represent mean ± s.d. (*n =* 3).

Considering the literature, the stability of SeNPs coated with different stabilizers was assessed in biological media by monitoring changes in size, PdI, and ζ‐potential. SeNPs were incubated in Roswell Park Memorial Institute 1640 Medium (RPMI), supplemented with 10% fetal bovine serum (FBS), or in human plasma at 37 °C for 2 hours (**Figure** **5**).

Initial observations indicated an increase in size and PdI observed for all positively and negatively charged SeNPs exhibiting different trends. BSA1‐SeNPs demonstrated a slight increase in size, particularly when incubated in plasma, followed by a decrease over time, suggesting reversible protein corona formation (Figure 5A,B). This observation was consistent with the fact that the protein content in the plasma is ≈20 times higher than in the cell culture medium and that FBS can improve the colloidal stability of positively charged NPs.^[^ ^56^ ^]^ However, BSA12‐SeNPs size increased over time, indicating instability (Figure 5C,D).

Chitosan‐SeNPs exhibited similar trends to BSA1‐SeNPs but with larger diameters in culture medium, suggesting destabilization by medium compounds (Figure 5E,F), such as phosphate ions, which are 5‐fold more concentrated in the RPMI medium than in the human plasma.^[^ ^56^ ^]^ Negatively charged SeNPs, PMVEMA‐SeNPs and TPGS‐SeNPs, showed stability profiles resembling positively charged SeNPs, indicating soft and hard corona formation despite their negative charge (Figure 5G–J).

**Figure 6:**
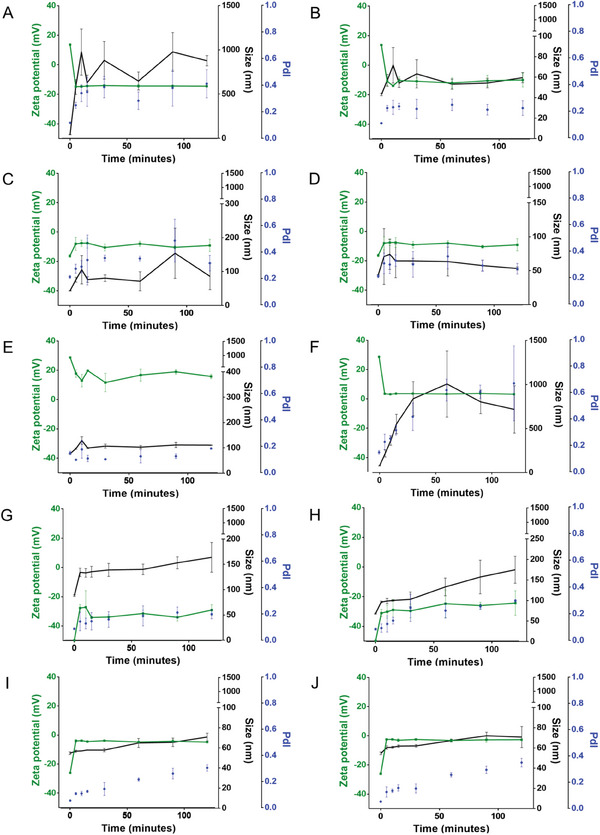
A,C,E,G,I) Stability profiles of SeNPs in MES (pH 5.5) and B,D,F,H,J) HEPES (pH 7.4) at 37 °C, for 2 hours: A,B) BSA1‐SeNPs; C,D) BSA12‐SeNPs; E,F) Chitosan‐SeNPs; G,H) PMVEMA‐SeNPs; I,J) TPGS‐SeNPs. Analysis of SeNP size (black), PdI (blue), and ζ‐potential (green). Error bars represent mean ± s.d. (*n =* 3).

NPs aimed for cancer treatment must be stable at physiologic pH (7.4) and degrade under the acidic conditions (≈5.5) characteristic of the TME.^[^ ^11^ ^]^ To investigate the pH‐responsiveness of the prepared NPs, we studied their stability in two aqueous buffers, 2‐(N‐morpholino)ethanesulfonic acid (MES) (1 M, pH 5.5) and 4‐(2‐hydroxyethyl)−1‐piperazineethanesulfonic acid (HEPES) (1 M, pH 7.4), to mimic the tumor and the physiologic pH environment, respectively (**Figure** **6**; Figure S6, Supporting Information). Long‐term stability studies revealed differences in size distribution between pH conditions, with BSA‐SeNPs exhibiting higher stability at physiologic pH and Chitosan‐SeNPs showing stability in acidic conditions. Overall, these findings underscore the potential of BSA1‐SeNPs for systemic administration, as they are the only type of SeNPs that remained stable under physiological conditions, in contrast to the precipitation observed for Chitosan‐SeNPs, PMVEMA‐SeNPs, and TPGS‐SeNPs, indicating their limited potential for use in animal studies.

### 2.5 SeNPs In Vitro Studies

SeNPs have garnered attention for potential use in breast cancer treatment, both in vitro and in vivo, either coated with targeting ligands like folic acid,^[^ ^57^ ^]^ other anticancer compounds such as calcium sulfate,^[^ ^58^ ^]^ or in combination with antiproliferative drugs like fluorouracil,^[^ ^59^ ^]^ epirubicin,^[^ ^60^ ^]^ or doxorubicin.^[^ ^61^ ^]^ Similarly, the therapeutic potential of SeNPs for lung cancer has been explored through functionalization with hyaluronic acid,^[^ ^62^ ^]^ and cyclic peptides, and by loading with doxorubicin^[^ ^63^ ^]^ or paclitaxel.^[^ ^64^ ^]^ However, there has not been a comprehensive comparison of the antitumor potential of SeNPs produced using different stabilizers. Although several stabilizers and functionalization agents are being used for antitumor SeNP production,^[^ ^16^ ^]^ a comparative study focusing on these differences is crucial to identify the most suitable stabilizers for producing potential clinically viable anticancer SeNPs.

**Figure 7:**
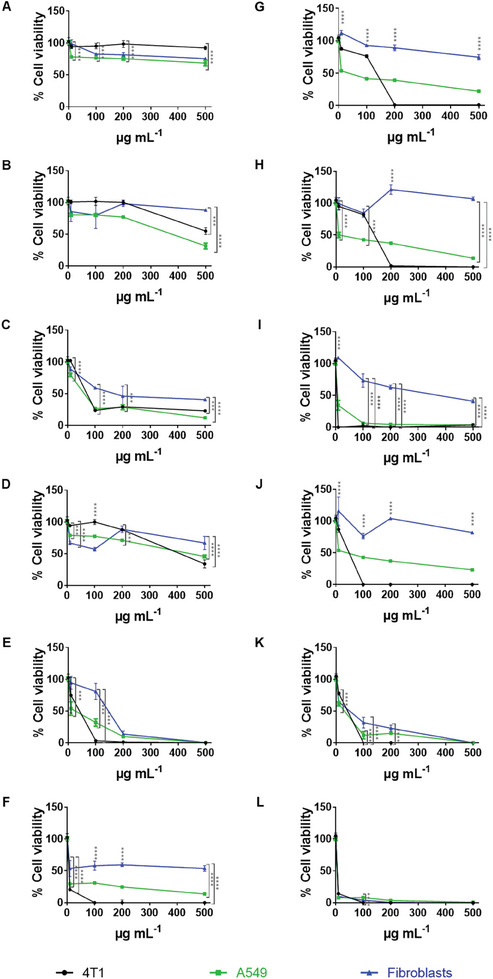
Antiproliferative studies of 4T1, A549 and human fibroblast cells treated with A,G) BSA1‐SeNPs, B,H) BSA12‐SeNPs, C,I) Chitosan‐SeNPs, D,J) PMVEMA‐SeNPs, E,K) TPGS‐SeNPs, and F,L) sodium selenite. Cells were incubated with different concentrations (10, 100, 200, and 500 µg mL^−1^) of SeNPs and sodium selenite prepared in complete medium, at 37 °C for A–F) 24 and G–L) 48 hours. Cells in complete medium were used as control. Data are presented as mean ± s.d. (*n* = 3). Statistical significance between the difference in viability among the cell lines studied for each concentration and time point (24 and 48 hours) was analyzed by two‐way analysis of variance (ANOVA) followed by Bonferroni post‐hoc test. The levels of significance were set at the probabilities of ^****^ *p* < 0.0001.

Several studies have reported the antiproliferative effects of SeNPs in lung and TNBC cells.^[^ ^57^, ^58^, ^63^ ^]^ Therefore, we investigated the anticancer properties of stabilized SeNPs using 4T1 and A549 cells as models for TNBC and lung cancer, respectively. In addition, primary human dermal fibroblasts were included to assess off‐target toxicity. We compared the anticancer potential of SeNPs stabilized with different compounds, including polymers, polysaccharides, and proteins while evaluating their toxicity in normal cells. Furthermore, we studied the cytotoxicity of sodium selenite to confirm the improved biocompatibility of SeNPs compared to their Se salt counterpart.^[^ ^16^ ^]^ Cytotoxicity assays were conducted using concentrations ranging from 10 to 500 µg mL^−1^ at 24 (**Figure** **7**A–F) and 48 hours (Figure 7G–L). The half‐maximal inhibitory concentration (IC_50_) values were determined for all cell lines and time points (Table S3, Supporting Information). As a control, the IC_50_ values of pure materials used as SeNP stabilizers (BSA; Chitosan; PMVEMA, and TPGS) were also determined (Table S4, Supporting Information).

Especially after 48 hours of incubation, sodium selenite (Figure 7L) showed higher toxicity than SeNPs for both cancer cells and fibroblasts (Figure 7G–K), even at the lowest concentration tested. SeNPs presented higher biocompatibility and tended to induce greater toxicity in cancer cells than in fibroblasts, supporting that SeNPs have higher biocompatibility compared to inorganic Se compounds.^[^ ^16^ ^]^ While at 24 hours, cancer cell viability was not significantly different from fibroblast viability (Figure 7A–E), after 48 hours, most SeNPs exhibited significantly higher toxicity toward cancer cells compared to fibroblasts (Figure 7G–K). Notably, TPGS‐SeNPs showed high cytotoxicity in both fibroblasts and cancer cells (Figure 7E,K). Furthermore, BSA‐SeNPs (Figure 7A,B,G,H) and PMVEMA‐SeNPs (Figure 7D,J) did not affect fibroblast viability, even at the highest concentration. PMVEMA‐SeNPs presented lower IC_50_ values for lung cancer (15.7 µg mL^−1^) compared to 4T1 breast cancer cells (48.0 µg mL^−1^), as did BSA1‐SeNPs (113.8 and 19.9 µg mL^−1^ for breast and lung cancer cells, respectively) and BSA12‐SeNPs (122.0 µg mL^−1^ for breast cancer cells, while the IC_50_ value for A549 was below the minimum concentration tested) (Table S3, Supporting Information). TPGS‐SeNPs and Chitosan‐SeNPs presented the highest toxicity for both fibroblasts and cancer cells, especially after 48 hours (Table S3, Supporting Information).

SeNPs showed distinct trends in inhibiting 4T1 and A549 cells, particularly after 48 hours, and depending on the stabilizer used (Figure 7G–K). While A549 cell viability decreased with increasing SeNP concentrations, 4T1 cell viability remained unaffected up to a certain concentration, beyond which SeNPs induced a significant reduction in cellular viability (Figure 7G–K). This underscores the influence of stabilizers on SeNPs' anticancer potential and antiproliferative effects. Although 6‐coumarin‐labeled BSA1‐SeNPs (6‐coumarin‐SeNPs) were internalized to a greater extent by 4T1 TNBC and EO771 luminal B breast cancer cells when compared to fibroblasts (*p* < 0.0001) (Figure S7, Supporting Information), their viability profiles were different (Figure S8, Supporting Information). EO771 cell viability showed a trend similar to A549 (Figure S8, Supporting Information), possibly indicating greater resistance to the SeNPs treatment than 4T1 cells.

Our findings suggest that the antitumor potential and toxicity of SeNPs to healthy cell lines are highly dependent on the stabilizer used. BSA‐SeNPs, PMVEMA‐SeNPs, and Chitosan‐SeNPs presented anticancer properties, especially at 48 hours, in concentrations that did not affect fibroblast viability, without the need for targeting agents or chemotherapeutic loading (Table S3, Supporting Information). SeNPs demonstrated time‐dependent effective anticancer properties at non‐toxic concentrations for healthy cells, highlighting their biocompatibility and effectiveness compared to inorganic Se compounds.

### 2.6 Therapeutic Impact of SeNPs on EO771 Tumor Growth

**Figure 8:**
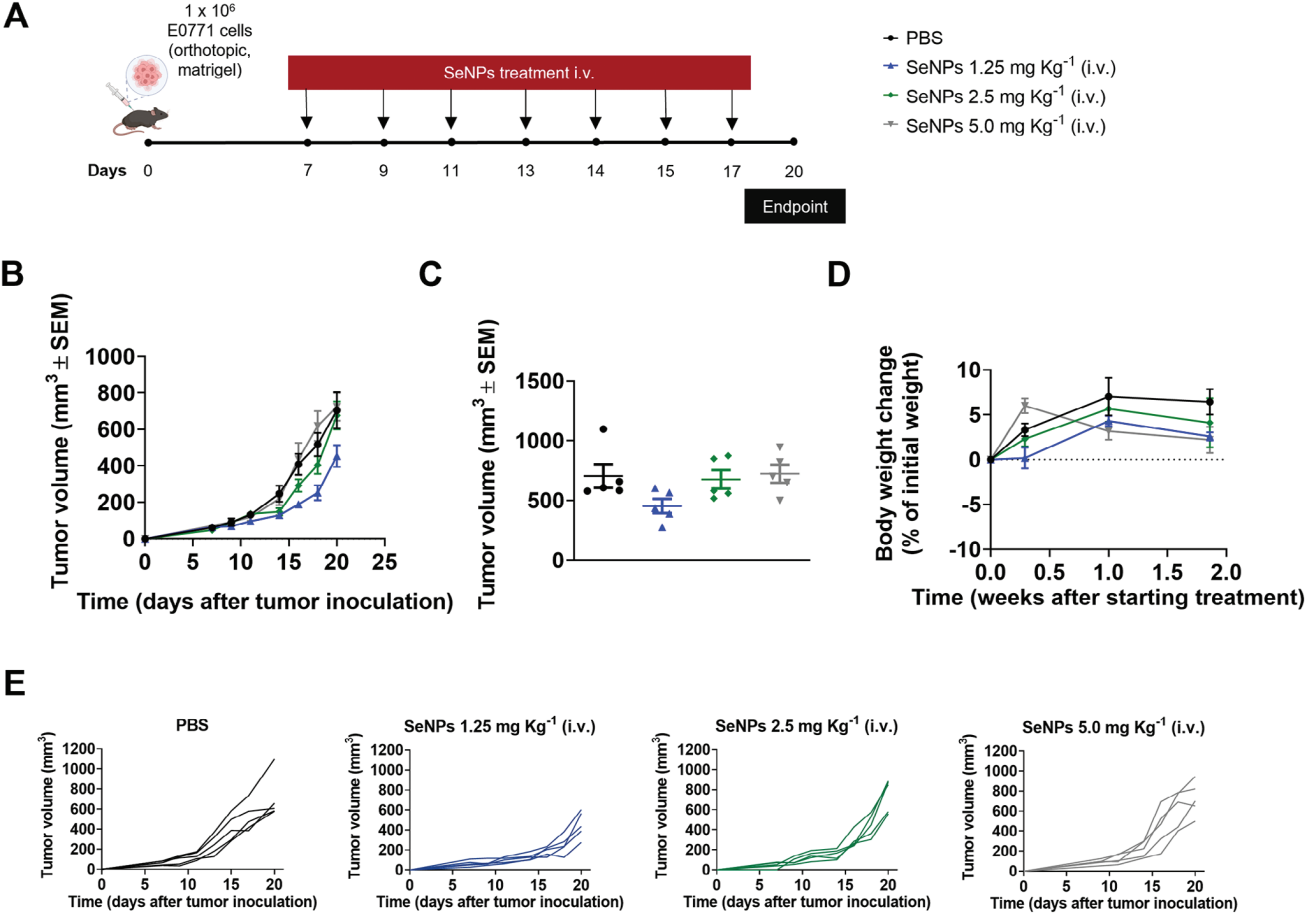
Intravenous administration of BSA1‐SeNPs induced concentration‐independent antitumor efficacy. A) C57BL/6J mice were orthotopically inoculated with 1 × 10^6^ EO771 tumor cells and treated with BSA1‐SeNPs at dosages of 1.25, 2.5, and 5 mg Kg^−1^ every 2 days. B) Average EO771 tumor growth curves. The data are presented as mean ± s.e.m of EO771‐bearing mice (n = 5 animals). C) Individual EO771 tumor volumes at day 20 following tumor inoculation. Statistical significance was analyzed by one‐way ANOVA followed by Tukey multiple comparisons post‐hoc test. D) Body weight change is expressed as the percent change in weight from the day of treatment initiation. E) Individual tumor growth curves.

The cytotoxic potential of the SeNPs was validated using the luminal B breast cancer EO771 cell line to assess the antitumor effect in a breast cancer orthotopic mouse model. BSA1‐SeNPs were selected for the in vivo study, due to their potent in vitro cytotoxic effect against cancer cells while sparing normal cancer cells. Moreover, BSA1‐SeNPs exhibited superior stability in human plasma and physiological pH compared to other SeNPs. The antitumor efficacy of BSA1‐SeNPs administered intravenously (i.v.) was evaluated at doses of 1.25, 2.5, and 5 mg Kg^−1^ every 2 days in EO771‐bearing mice, following the schedule in **Figure** **8**A.

Despite increasing the dose of BSA1‐SeNPs, tumor growth was not effectively controlled. Mice treated with BSA1‐SeNPs at 1.25 mg Kg^−1^ showed slightly smaller tumor volumes (Figure 8B), although these differences were not statistically significant compared to the phosphate‐buffer saline (PBS)‐treated mice (Figure 8C). This suggests that the intravenous administration of SeNPs for anticancer purposes may require the presence of a targeting ligand, such as folic acid, hyaluronic acid, or arginylglycylaspartic acid peptide.^[^ ^16^ ^]^

However, the administration of all doses of BSA1‐SeNPs presented minimal body weight changes (Figure 8D), suggesting their systemic safety for parental administration also corroborated by the absence of haemolysis in mouse red blood cells incubated with the BSA1‐SeNP (Table S5, Supporting Information).

### 2.7 SeNPs Synergize with a *wild‐type* Kirsten rat sarcoma viral oncogene homologue (KRAS_*wt*_) Nanovaccine against EO771 Tumors

Considering the intricate nature of the immune response within the breast cancer microenvironment, there is an urgent need to develop advanced combination therapies that target multiple pathways, reinforcing, reprogramming, and restoring robust antitumor innate and adaptive immune responses, while also inducing direct cancer cell death. This approach is crucial to combat therapeutic resistance and prolong the survival of breast cancer patients.

One promising strategy is cancer vaccination, which aims to re‐educate the patient's T‐cell responses, leading to antigen‐directed cell death and the generation of a memory response to prevent disease progression.^[^ ^65^, ^66^, ^67^ ^]^ Although single therapy using BSA1‐SeNPs i.v. did not significantly reduce the tumor growth in EO771‐bearing mice, it did exhibit some delay. To enhance the therapeutic efficacy, we proposed a combination therapy involving a therapeutic nanovaccine delivering the ***wild‐type*** Kirsten rat sarcoma viral oncogene homologue (KRAS_*wt*_) antigen along with BSA1‐SeNPs, administered either intratumorally (i.t.) or i.v. at a dose of 1.25 mg kg^−1^.

KRAS_*wt*_ is associated with the expression of HER2, usually overexpressed in breast cancer, being also linked to the development of TNBC, enabling immune evasion.^[^ ^68^ ^]^ Therefore, we hypothesized that the combined treatment of BSA1‐SeNPs and KRAS_*wt*_ nanovaccine could result in the modulation of systemic and tumor‐infiltrating immune‐cell subpopulations, resulting in a stronger EO771 tumor growth control.

**Figure 9:**
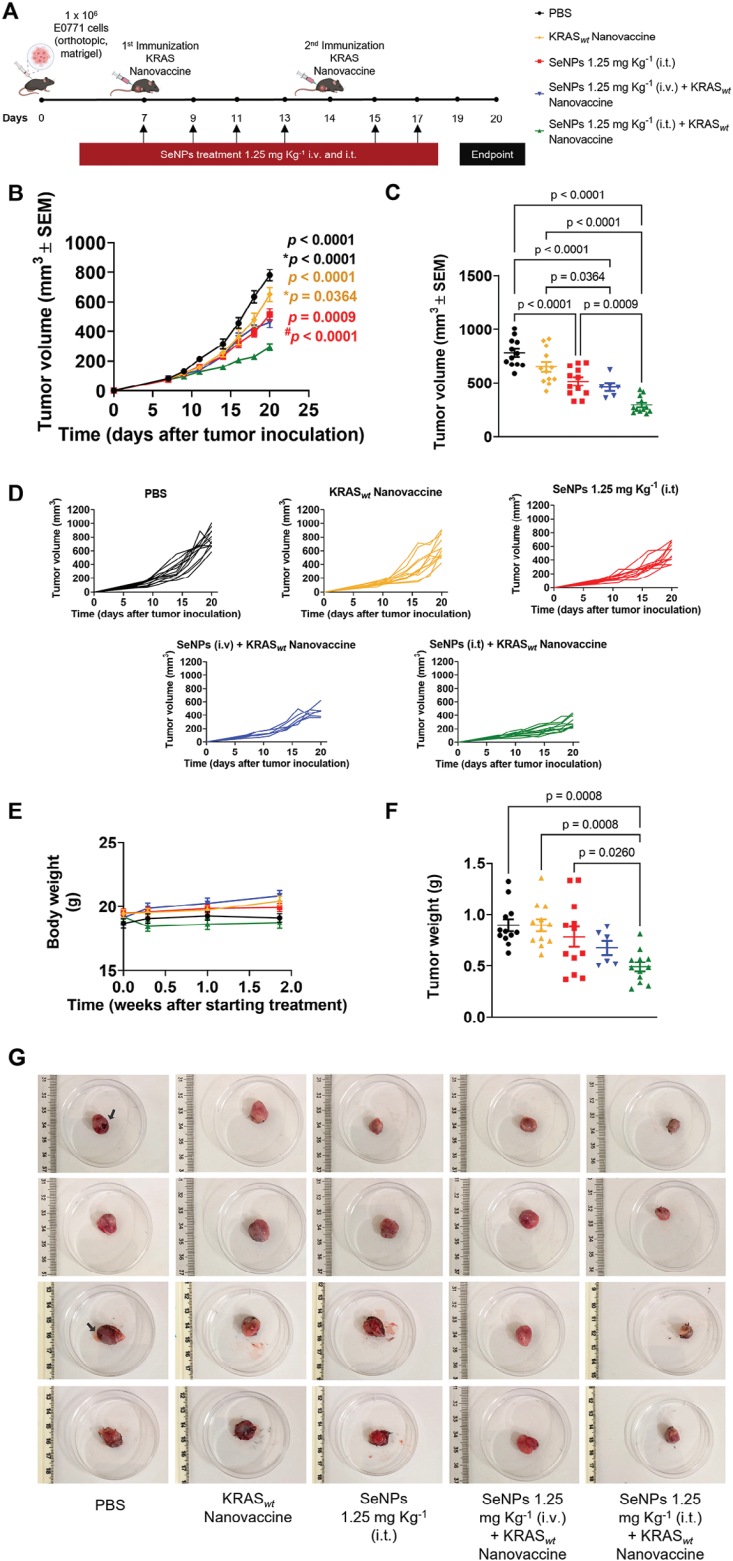
A combination schedule of KRAS_*wt*_ nanovaccine and BSA1‐SeNPs controlled the aggressive tumor growth of EO771 luminal B breast cancer. A) C57BL/6J mice were orthotopically inoculated with 1 × 10^6^ EO771 tumor cells and treated with BSA1‐SeNPs every other day and KRAS_*wt*_ nanovaccine on days 7 and 14. B) Average EO771 tumor growth curves. Data are presented as mean ± s.e.m of EO771‐bearing mice (*N* = 2 in vivo assays, *n* = 6 animals per assay for all treatment groups except for SeNPs 1.25 mg Kg^−1^ (i.v.) + KRAS_*wt*_ Nanovaccine (*n* = 6)). Statistical significance was analyzed by one‐way ANOVA followed by Tukey multiple comparisons post‐hoc test and *p*, p*, and *p^#^* values correspond to tumor volume at day 20 after tumor inoculation, relative to SeNPs 1.25 mg Kg^−1^ (i.t.) + KRAS_*wt*_ Nanovaccine, SeNPs 1.25 mg Kg^−1^ (i.v.) + KRAS_*wt*_ Nanovaccine, and SeNPs 1.25 mg Kg^−1^ (i.t.), respectively. C) Individual EO771 tumor volumes at day 20 following tumor inoculation. Statistical significance was analyzed by one‐way ANOVA followed by Tukey multiple comparisons post‐hoc test and *p* values correspond to tumor volume at day 20 after tumor inoculation. D) Individual tumor growth curves. E) Body weight (g) from the day of treatment initiation. F) Individual EO771 tumor weights at day 20 following tumor inoculation. Data are presented as mean ± s.e.m of EO771‐bearing mice (*N* = 2 in vivo assays, *n* = 6 animals per assay for all treatment groups except for SeNPs 1.25 mg Kg^−1^ (i.v.) + KRAS_*wt*_ Nanovaccine (*n* = 6)). Statistical significance was analyzed by one‐way ANOVA followed by Tukey multiple comparisons post‐hoc test and *p* values correspond to tumor weight at day 20 after tumor inoculation. G) Representative images of tumors at day 20.

KRAS_*wt*_ nanovaccine was administered on days 7 and 14, in combination with BSA1‐SeNPs, following the schedule outlined in **Figure** **9**A. Mice treated with the combination SeNPs 1.25 mg Kg^−1^ (i.t.) + KRAS_*wt*_ Nanovaccine presented significantly smaller tumor volumes on day 20 compared to other treatment groups, including PBS (*p* < 0.0001), KRAS_*wt*_ Nanovaccine (*p* < 0.0001), and SeNPs 1.25 mg Kg^−1^ (i.t.) (*p* = 0.0009) (Figure 9B–D). Although this treatment induced lower tumor volumes compared to SeNPs 1.25 mg Kg^−1^ (i.v.) + KRAS_*wt*_ Nanovaccine group (Figure 9B–D), no significant differences were observed. Moreover, this combination therapy induced negligible changes in body weight, indicating its systemic safety for parenteral administration (Figure 9E).

Analysis of tumor volume and weight further supported the synergistic effect between KRAS_*wt*_ nanovaccine and BSA1‐SeNPs (i.t.), with this combination yielding the lowest average tumor volume (295 mm^3^, Figure 9C) and average tumor weight (0.49 g, Figure 9F) compared to other treatment modalities. Indeed, the synergistic effect of KRAS_*wt*_ nanovaccine combined with SeNPs could be confirmed by the 62.2% of tumor growth inhibition compared to 34% and 16.6% for SeNPs (i.t.) and KRAS_*wt*_ nanovaccine, respectively. Furthermore, images of tumors at day 20 revealed necrosis in PBS‐treated mice (black arrow), which was absent in tumors from mice receiving single or combined treatments (Figure 9G).

**Figure 10:**
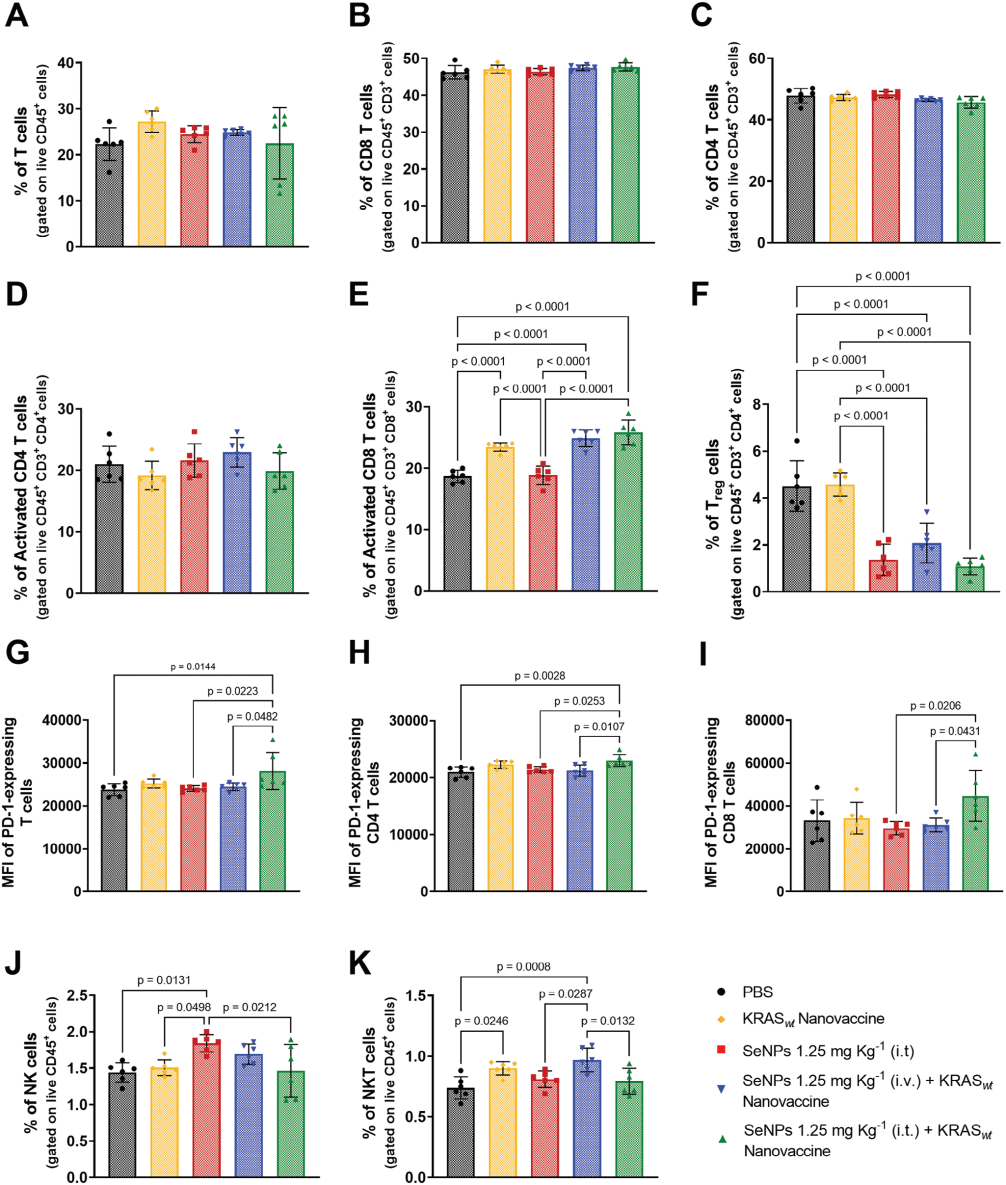
Systemic activation of the CD8^+^ T effector and decreased Treg levels by the divalent combination of SeNPs intratumorally administrated with the KRAS_*wt*_ nanovaccine. A–K) Percentage of the systemic CD3^+^ T (A), CD8^+^ T (B), CD4^+^ T (C), effector activated CD4^+^ T (D), effector activated CD8^+^ T (E), Treg (F), PD‐1‐expressing T (G), PD‐1^+^‐expressing CD4^+^ T (H), PD‐1^+^‐expressing CD8^+^ T (I), NK (J), and NKT (K) cells. Spleens were recovered on day 20 following tumor inoculation. The quantification was performed by flow cytometry analysis. Data are presented as mean ± s.d., n = 6 animals. Statistical significance was calculated by one‐way ANOVA with Tukey multiple comparisons post‐hoc test.

The tumors and spleens of the treated mice were analyzed by flow cytometry according to the panels described in Figures S9 and S10 (Supporting Information). Although no significant differences have been observed for general T cells (**Figure** **10**A), including CD8^+^ T (Figure 10B), CD4^+^ T (Figure 10C), and activated CD4^+^ T (Figure 10D) cells, the combined therapy SeNPs 1.25 mg Kg^−1^ (i.t.) + KRAS_*wt*_ Nanovaccine increased the systemic levels of activated CD8^+^ T cells (*p* < 0.0001) (Figure 10E). Furthermore, the treatment with BSA1‐SeNPs, as single therapy or combined with the KRAS_*wt*_ nanovaccine, reduced the systemic levels of Treg cells when compared to PBS‐ and KRAS_*wt*_ nanovaccine‐treated groups (*p* < 0.0001) (Figure 10F). An enhanced level of systemic T cells expressing PD‐1 (Figure 10G), including both PD1‐expressing CD4 (Figure 10H) and CD8 (Figure 10I) cells, were also observed in mice treated with the combination of BSA1‐SeNPs (i.t.) and KRAS_*wt*_ nanovaccine when compared to PBS‐, SeNPs 1.25 mg Kg^−1^ (i.t.)‐, and SeNPs 1.25 mg Kg^−1^ (i.v.) + KRAS_*wt*_ Nanovaccine‐treated groups (*p* < 0.05). Moreover, the highest levels for NK and NKT cells were observed for the mice treated with SeNPs 1.25 mg Kg^−1^ (i.t.) (*p* = 0.0131) (Figure 10J) and SeNPs 1.25 mg Kg^−1^ (i.v.) + KRAS_*wt*_ Nanovaccine (*p* = 0.0008) (Figure 10K), respectively.

**Figure 11:**
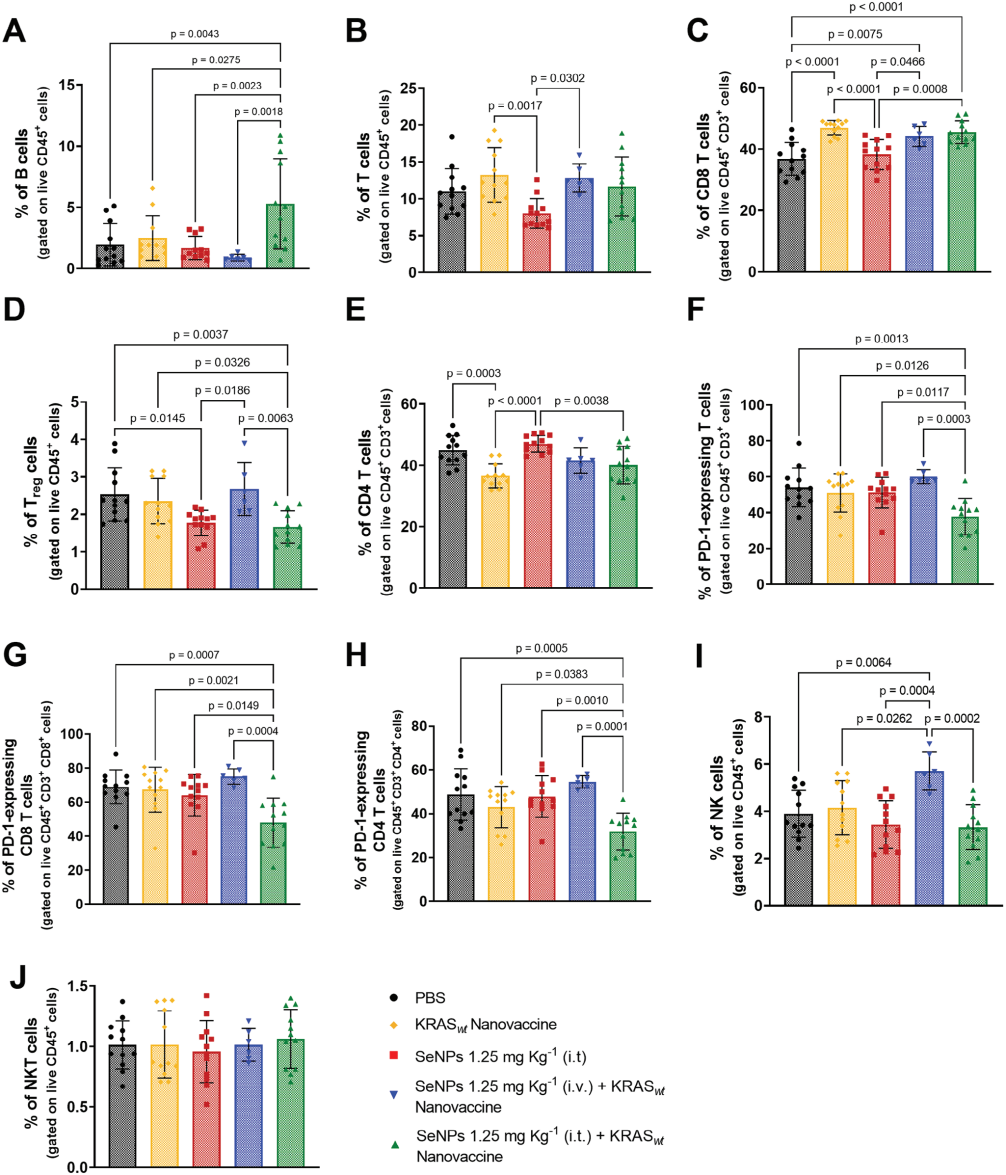
Highest infiltration of B and CD8^+^ T cells, and decreased Treg and PD1‐expressing T cell levels for divalent combination of SeNPs intratumorally administered with KRAS_*wt*_ nanovaccine. A–J) Tumor‐infiltrating immune cell populations for B (A), CD3^+^ T (B), PD‐1^+^‐expressing T (C), CD8^+^ T (D), PD‐1^+^‐expressing CD8^+^ T (E), CD4^+^ T (F), PD‐1^+^‐expressing CD4^+^ T (G), Treg (H), NK (I), and NKT (J) cells. Tumors were recovered on day 20 following tumor inoculation. The quantification was performed by flow cytometry analysis. Data are presented as mean ± s.d., *N* = 2 in vivo assays, *n* = 6 animals per assay for all treatment groups except for SeNPs 1.25 mg Kg^−1^ (i.v.) + KRAS_*wt*_ Nanovaccine (*n* = 6)). Statistical significance was calculated by one‐way ANOVA with Tukey multiple comparisons post‐hoc test.

Additionally, the combined therapy SeNPs 1.25 mg Kg^−1^ (i.t.) + KRAS_*wt*_ Nanovaccine boosted the infiltration of B cells (**Figure** **11**A) in the TME compared to the PBS (*p* = 0.0043), KRAS_*wt*_ Nanovaccine (*p* = 0.0275), SeNPs 1.25 mg Kg^−1^ (i.t.) (*p* = 0.0023) and the SeNPs 1.25 mg Kg^−1^ (i.v.) + KRAS_*wt*_ Nanovaccine (*p* = 0.0018) groups. B cell abundance is positively correlated with better clinical outcomes since B cells are involved in the presentation of cancer antigens, and activation of CD8^+^ T cells, and the release of antibodies and cytokines, inducing the humoral immunity.^[^ ^69^ ^]^ Therefore, this promising divalent therapy SeNPs 1.25 mg Kg^−1^ (i.t.) + KRAS_*wt*_ Nanovaccine boosted T‐cell infiltration into tumors (Figure 11B), resulting in high levels of intratumoral CD8^+^ T cells (*p* < 0.0001) (Figure 11C). The prominent infiltration of CD8^+^ T cells within the tumors on day 20 in the SeNPs 1.25 mg Kg^−1^ (i.t.) + KRAS_*wt*_ Nanovaccine group was further confirmed by immunohistochemistry staining of EO771 tumor sections for CD8 (Figure S11, Supporting Information), thus corroborating the flow cytometry results.

The divalent combination of SeNPs intratumorally administered with KRAS_*wt*_ nanovaccine also reduced tumor‐infiltrating Treg cells (*p* = 0.0012) (Figure 11D), a subset of CD4^+^ T cells (Figure 11E), which, due to their immunosuppressive properties, prevent tumor immunosurveillance and response, contributing to its progression.^[^ ^70^ ^]^ Moreover, significant decreased levels of tumor‐infiltrating PD1‐expressing T cells (Figure 11F), including both PD1‐expressing CD8 (Figure 11G) and CD4 (Figure 11H) cells, were also observed in the tumors of these animals when compared to groups treated with PBS (*p* < 0.01), KRAS_*wt*_ Nanovaccine (*p* < 0.05), SeNPs 1.25 mg Kg^−1^ (i.t.) (*p* < 0.05), and the SeNPs 1.25 mg Kg^−1^ (i.v.) + KRAS_*wt*_ Nanovaccine (*p* < 0.001). It is also possible to observe an increased infiltration of NK cells in the TME, however only for the group SeNPs 1.25 mg Kg^−1^ (i.v.) + KRAS_*wt*_ Nanovaccine (*p* = 0.0064) (Figure 11I), which corroborates the studies indicating that SeNPs can enhance NK cells antitumor response.^[^ ^71^ ^]^ No significant differences were observed between treated groups for tumor‐infiltrating NKT cells (Figure 11J).

This superior antitumor effect was also reinforced by the tolerability and safety of the nano‐based divalent combination of SeNPs intratumorally administrated with the KRAS_*wt*_ nanovaccine, demonstrated by the absence of acute toxicity signs (Figure S12, Supporting Information). On day 20 following tumor inoculation, the biochemical analysis of murine blood showed basal levels for the activity of the liver function enzymes, alanine aminotransferase (ALT) (Figure S12A, Supporting Information), gama glutamil transferase (GGT) (Figure S12B, Supporting Information), and alkaline phosphatase (Figure S12C, Supporting Information) for all treatment groups. No alterations were observed for creatinine levels (Figure S12D, Supporting Information) in response to all treatments. The physiologic liver and kidney functions are also supported by the basal total proteins (Figure S12E, Supporting Information) and albumin (Figure S12F, Supporting Information) levels. In addition, no differences were obtained for total cholesterol levels (Figure S12G, Supporting Information) suggesting no cardiovascular dysfunction caused by treatments. No histological differences were observed among mono and nano‐based divalent regimens (Figure S13, Supporting Information). Multifocal small foci of inflammatory cell infiltration forming mononuclear microgranulomas were observed in the liver of animals from all groups with no clear associated hepatocyte cell death (Figure S13, Supporting Information). Minimal tubular mineralization in the renal papillae was found as a spontaneous lesion (background finding) in the kidneys of animals from all groups, with no clinical relevance (Figure S13, Supporting Information). No significant alterations (within normal limits) were detected in the heart and spleen (Figure S13, Supporting Information). Overall, this study validated our hypothesis that intratumorally administrated BSA1‐SeNPs act as an immunomodulator by confirming the synergism with the KRAS_*wt*_ nanovaccine, which translated into a strong tumor growth control of the EO771 luminal B breast cancer mouse model, thus supporting the potential application of our divalent approach as an efficient and safe therapy against solid tumors.

## 3 Conclusion

Here, the production of different types of SeNPs was optimized. We observed that, depending on the stabilizer used during the preparation, different parameters influenced the physiochemical properties of SeNPs. These parameters included the need and temperature of sonication, the amount of ascorbic acid, sodium selenite_,_ and BSA used, the flow rate for ascorbic acid addition to the mixture of sodium selenite and stabilizer, as well as the reaction time. The SeNPs produced were also stable over time, especially at 4 °C. BSA1‐SeNPs proved to be the most stable, preserving the size, PdI, and ζ‐potential for 3 months both at room temperature and 4 °C, as well as after resuspension following freeze‐drying. All other SeNPs required the use of a cryoprotectant, such as sucrose or trehalose, to maintain their characteristics during the freeze‐drying process. BSA1‐SeNPs presented pH‐responsive properties, degrading under acidic conditions mimicking the TME, while remaining stable under pH 7.4, indicating their ability to evade opsonization and subsequent phagocytic elimination while in blood circulation. Furthermore, BSA1‐SeNPs, BSA12‐SeNPs, Chitosan‐SeNPs, and PMVEMA‐SeNPs presented antiproliferative properties in breast and lung cancer cell lines, without causing major toxicity for fibroblasts, especially after 48 hours of incubation. This suggests that these SeNPs presented anticancer properties without adversely affecting the viability of healthy cells. Overall, considering the physicochemical properties of SeNPs, their stability in a biological medium, and their impact on the viability and proliferation of cancer cells while preserving the biological functions of healthy cells, BSA‐SeNPs exhibit strong potential for biomedical applications, such as anticancer agents. Furthermore, BSA1‐SeNPs also demonstrated antitumor properties in EO771‐bearing mice, effectively controlling tumor growth when combined with a KRAS_*wt*_ nanovaccine. This combination increased both systemic and intratumoral levels of activated CD8^+^ T cells, reduced levels of Treg cells, and enhanced infiltration of B cells in the TME, demonstrating the potential of BSA1‐SeNPs for cancer immunotherapy.

In conclusion, the remarkable antitumor efficacy of BSA1‐SeNPs, coupled with their ability to modulate the immune microenvironment by enhancing CD8+ T cell activation and reducing immunosuppressive cell populations, underscores their promising role in cancer immunotherapy as a multifaceted therapeutic agent.

## 4 Experimental Section

### Reagents

MES, HEPES, acetic acid, L(+)‐ascorbic acid, BSA, chitosan (molecular weight (M_W_) = 50 – 190 KDa), PMVE‐MA (M_W_ = 1,980 KDa), sodium selenite, sucrose, D‐(+)‐trehalose dihydrate, dichloromethane (DCM), D‐alpha‐tocopherol polyethylene glycol 1000 succinate (TPGS), pluronic F‐127 (PF127), trypan blue solution 0.4%, liquid, sterile‐filtered, suitable for cell culture. Chitosan (M_W_ = 15 KDa) and Poly(L‐lactic acid) (PLA, M_w_ 1600 – 2400, viscosity 0.15 dl g^−1^) was obtained from Polysciences, Inc. Sodium Hydroxide (NaOH) and Hydrochloric acid (HCl) were purchased from VWR. Sodium borohydride (NaBH_4_) was acquired from Acros Orcanics. Nitric acid (HNO_3_) was purchased from Romil, Super purity. Major histocompatibility complex (MHC) II‐restricted KRAS_*wt*_ peptide: LT‐16 was purchased from Genecust. Cytosine phosphorothioate‐guanine motifs (CpG)‐oligodeoxynucleotides (ODN) 1826 (TCCATGACGTTCCTGACGTT) was purchased from Microsynth GmbH. Poly(I:C) (High M_w_) VacciGrade was purchased from Invitrogen.

High glucose Dulbecco's Modified Eagle medium (DMEM), RPMI, L‐glutamine (200 mm), non‐essential amino acids (NEAA), heat inactivated FBS, trypsin‐ethylenediaminetetraacetic acid (trypsin‐EDTA 0.25%), pierce 16% paraformaldehyde (m/v), PBS, pH 7.4, penicillin‐streptomycin (PEST; penicillin 10 000 U ml^−1^ and streptomycin 10 000 mg mL^−1^), sodium pyruvate (100 mm), HEPES buffer solution (1 m), and ammonium‐chloride‐potassium (ACK) lysing buffer were acquired from Thermo Fisher Scientific. Hank's balanced salt solution (HBSS) was purchased from Life Technologies Gibco. CellTiter‐Glo reagent assay was purchased from Promega Corporation. Plasma was obtained from anonymous blood donors (B‐) from the Finnish Red Cross Service. Collagenase type II (LS004176); neutral protease (LS02109); and deoxyribonuclease I (DNAse I; LS002006) were purchased from Worthington Biochemical Corporation (Freehold, NJ, USA).

### Cell Culture

Primary human dermal fibroblasts, kindly provided by Dr. Jackson, Biomedicum, University of Helsinki, were cultured in high glucose DMEM supplemented with 10% (v/v) FBS, 1% (v/v) NEAA, 1% (v/v) L‐glutamine, 1% (v/v) PEST, and 1% (v/v) sodium pyruvate. A549 (human lung carcinoma), 4T1 (TNBC murine cell line), and EO771 (luminal B breast cancer murine cell line) cells were obtained from the American Type Culture Collection (ATCC). A549 cells (ATCC CCL‐185) were cultured in DMEM high glucose supplemented with 10% (v/v) FBS, 1% (v/v) NEAA, 1% (v/v) L‐glutamine, and 1% (v/v) PEST. 4T1 cells (ATCC CRL‐2539) were cultured in RPMI supplemented with 10% (v/v) FBS, 1% (v/v) NEAA, 1% (v/v) L‐glutamine and 1% (v/v) PEST. EO771 cells (ATCC CRL‐3461) were cultured in DMEM high glucose + pyruvate medium supplemented with 10% (v/v) FBS, 1% (v/v) PEST and 2% (v/v) HEPES. Cells were maintained in an incubator at 37 °C equilibrated with 5% CO_2_ and 95% relative humidity.

### Synthesis of SeNPs

SeNPs were prepared using sodium selenite and ascorbic acid as the selenium precursor and the reducing agent, respectively. The molar ratio of Se:ascorbic acid used for the preparation of SeNPs was 1:4. Two production methods were used for SeNPs preparation. In the first method, 5 mL of the stabilizer solution was mixed with 5 mL of sodium selenite. After, 5 mL of ascorbic acid was added at a consistent flow rate using a microfluidic pump (PHD 2000, Harvard Apparatus). In the second method, 8 mL of ascorbic acid was added to 1 mL containing sodium selenite and the stabilizer, also at a constant flow rate using a microfluidic pump. The formed SeNP suspension was dialyzed against fresh Milli‐Q water for 24 hours using a dialysis bag (Spectra/Por 1 Standard RC Dry Dialysis Tubing, Spectrum Labs). The following production parameters were optimized for each stabilizer: production methods; the amount of stabilizer and ratio sodium selenite/ascorbic acid; the temperature of sonication (Sonics VibraCell VCX‐750 Ultrasonic processor with stepped microtip 2 mm); the ascorbic acid flow rate; stirring speed; time of reaction after the addition of ascorbic acid, dialysis membrane pore size, and the temperature of dialysis. The impact of the M_w_ of chitosan and PMVE‐MA on SeNP properties was also studied. A PCA using the PCA module of the Sklearn python package was performed to better understand the effect of these variables on SeNP properties to select the optimal composition and preparation conditions for subsequent studies.

### Synthesis of KRAS_wt_ Nanovaccine

The double emulsion (w/o/w) solvent evaporation method was used for the preparation of PLA/poly(lactic‐co‐glycolic) acid (PLGA)‐Mannose (PLA‐Man) NP.^[^ ^72^ ^]^ Briefly, the PLA (8 mg) and PLGA‐Mannose (2 mg) were dissolved in DCM at 50 mg mL^−1^ (oil phase). The internal aqueous phase was prepared by dissolving first the toll‐like receptor ligands (CpG‐ODN at 1.4 mg mL^−1^ and Poly(I:C) at 2.8 mg mL^−1^) in 10% (v/v) poly(vinyl alcohol) aqueous solution, and finally, the KRAS_*wt*_ MHC II peptide (at 10 mg mL^−1^). The internal aqueous phase was added to the oil phase containing the dissolved polymer blends and the mixture was emulsified using sonication for 15 seconds at 20% amplitude (Digital Sonifier Cell Disruptor, Branson Ultrasonics, Emerson). A second emulsion was formed by adding TPGS aqueous solution under the same conditions. The w/o/w emulsion was added dropwise into a 0.125% (m/v) PF127 aqueous solution and stirred for 1 hour at room temperature. NPs were collected by centrifugation at 22 000 *g*, at 4 °C, for 40 minutes (Beckman Coulter Allegra 64R High‐Speed Centrifuge), and finally resuspended in PBS.

### Characterization of SeNPs

The average particle size (Z‐average), PdI, and average ζ‐potential (surface charge) of SeNPs were characterized using a Malvern Zetasizer Nano ZS instrument (Malvern Instruments Ltd). 100 µL SeNP samples were diluted in 900 µL MilliQ‐water. Optical properties of SeNPs samples were analyzed by UV spectroscopy using a UV/Visible spectrophotometer, UV‐1600 PC, and by ATR‐FTIR (Vertex 70, Bruker) to confirm the presence of Se and the stabilizer, respectively. The size distribution and the morphology of the different types of SeNPs were evaluated using a Jeol JEM‐1400 TEM microscope (Ltd.), equipped with a Gatan Orius SC 1000B bottom‐mounted CCD‐camera (Gatn Inc.) and a BF‐TE detector in a Hitachi S‐4800 field emission SEM. For sample preparation, 5 µL of each SeNP suspension was placed on a carbon‐coated copper grid, blotted using filter paper, and then air‐dried for 24 hours before analysis. EDS analysis was performed using an Oxford INCA 350 EDS spectrometer connected to the Hitachi S‐4800 SEM. A bright field transmission electron detector (BF‐TE) was used for acquiring the images where the EDS measurement areas were selected.

SeNPs selenium content was measured by microwave plasma atomic emission spectrometer (Agilent 4200 MP‐AES) equipped with a multimode sample introduction system for selenium hydride generation. For this measurement, 0.1% (m/v) NaBH_4_ solution stabilized in 0.5% (m/v) NaOH was used as a reducing agent. Wavelength 196.026 nm was used for the Se emission analysis.^[^ ^73^ ^]^ Before Se analysis, SeNPs were digested/decomposed using HNO_3_/H_2_O_2_: 0.1 mL of sample was pipetted in 4 mL of 65% HNO_3_ and was let to stand for 8 hours before adding 0.1 mL of 30% (v/v) H_2_O_2_. After standing for 16 hours, 0.5 mL subsamples were diluted with 4.5 mL of 6 m HCl and heated to 70 °C for 1 hour.^[^ ^73^ ^]^

The number of NPs in each SeNP suspension was quantified by nanoparticle tracking analysis using a NanoSight LM10, Malvern Panalytical, by diluting 10 µL of each SeNP suspension in 50 mL Milli‐Q water.

Elemental analysis was performed using a HANAU Elementar Analysensysteme GmbH, (Germany. vario MICRO cube. Serial no. 15082023). The protocol for SeNP separation is reported in Table S6 (Supporting Information).

### Storage and Stability Studies

*SeNP* stability was assessed in cell medium, human plasma, HEPES (1 m, 7.5 pH), and MES (1 m, pH 5.5). For that, 100 µL of SeNPs were incubated with 900 µL of the different solutions at 150 rpm at 37 °C. Samples (200 µL) were collected at 5, 10, 30, 90, and 120 minutes after incubation and diluted in 800 µL of Milli‐Q water to evaluate changes in SeNPs’ size over time. All experiments were performed in triplicate.

Long‐term stability assays were performed by mixing 2 mL of SeNPs with 8 mL of medium (HEPES pH 7.4, MES pH 5.5, and RPMI medium supplemented with 10% (v/v) FBS), which were left stirring at 150 rpm at 37 °C for one week. A visual assessment of the samples was carried out, documented by pictures, while the shape and size of the nanosystems were further imaged using a Hitachi S‐4800 Field Emission Scanning Electron Microscopy. In brief, 5 µL of SeNPs were dropped to a carbon‐coated copper grid (Electron Microscope FCF 200‐CU Mesh Copper) for 5 minutes, the excess was removed with filter paper and dried overnight.

The properties of SeNPs were also evaluated after freeze‐drying (Freeze drier Scanvac Coolsafe 110 – 4 Pro using a Vacuubrand RZ 2.5 vacuum pump), with and without cryoprotectants (trehalose and sucrose). Cryoprotectants were studied at 5, 10, 20, and 40 mg mL^−1^, in a final volume of 1.5 mL.

SeNPs storage stability was addressed by keeping SeNPs suspension at both room temperature and 4 °C, and further measuring the mean average size, PdI, and ζ‐potential, 12 weeks after storage.

### Antiproliferation Studies

4T1 murine cells, a metastatic phenotype of breast cancer similar to human TNBC,^[^ ^74^, ^75^ ^]^ EO771 luminal B breast cancer cells, a lung adenocarcinoma cell line A549,^[^ ^76^ ^]^ and primary human fibroblasts were seeded in 96‐well plates at a density of 20,000 cells per well and allowed to attach overnight. The medium was then removed, and cells were treated with SeNPs (100 µL) at different concentrations (10, 100, 200, and 500 µg mL^−1^). Plates were incubated at 37 °C at two different time points, 24 and 48 hours. Cell medium and 1% (v/v) Triton X‐100 were used as negative and positive controls, respectively. After that, plates were equilibrated at room temperature for 30 minutes and washed with 100 µL of HBSS–HEPES (pH 7.4) buffer. Then, 50 µL of HBSS–HEPES (pH 7.4) followed by 50 µL of CellTiter‐Glo were added to each well. Plates were stirred for 2–5 minutes in an orbital shaker and then stabilized for 15 minutes at room temperature, protected from light. Cell viability was quantified based on the adenosine triphosphate levels produced by metabolically active cells,^[^ ^77^ ^]^ using a Varioskan Flash plate reader (Thermo Fisher Scientific, Inc.). All experiments were performed at least in triplicate.

### In Vitro SeNP Internalization Studies

Coumarin‐6‐labeled BSA1‐SeNPs (coumarin‐6‐SeNPs) were prepared by mixing BSA1‐SeNPs (5 mL) with coumarin‐6 (4 µg mL^−1^) and stirring at 400 rpm for 24 hours, protected from light. Fibroblasts and 4T1 TNBC cells (2 × 10^4^ cells per well), and EO771 luminal B breast cancer cells (4.5 × 10^3^ cells per well) were seeded in 96‐well plates and incubated overnight. Cells were then incubated with coumarin‐6‐SeNPs (100 µg mL^−1^; 485/525 nm of excitation/emission wavelengths) for 3 hours. Cells were harvested by centrifugation, washed with PBS, and incubated with Ghost Dye Red 780 (Cytek Biosciences, Cat# 13‐0865‐T500, 1:10,000) for 15 miutes at room temperature. Cells were finally fixed using 2% (v/v) paraformaldehyde and washed. The individual fluorescence for each sample was assessed using a Cytek Aurora cytometer (Cytek) and FlowJo software version 7.6.5 for Microsoft (TreeStar).

### Animal Studies

Female C57BL/6J (9–14 weeks‐old) mice were purchased from Instituto Gulbenkian de Ciência (Portugal) and accommodated in the animal facility of the Faculty of Pharmacy at the University of Lisbon. All animal procedures were completed in compliance with the Faculty of Pharmacy, University of Lisbon guidelines. Protocols were reviewed and approved by the Direção‐Geral de Alimentação e Veterinária (DGAV Portugal). Animals were housed under a 12‐hour light, 12‐hour dark cycle, with food and water available *ad libitum* and handled in compliance with the National Institutes of Health guidelines and the European Union (EU) rules for the care and handling of laboratory animals (Directive 2010∖63∖EU). Mice body weight change was monitored three times a week. Mice were euthanized in case of distress signs or rapid weight loss (above 10% within a few days or 20% from the initial weight), according to ethical protocol. Tumor‐bearing mice were euthanized when the tumor volume surpassed 1000 mm^3^. Tumor volume (mm^3^) was measured every 2 days and determined by $\frac{d2*D}{2}$, where d and D were the shortest and longest diameter in mm, respectively.

### In Vivo Impact of SeNPs Concentration on the EO771 Tumor Growth

On day 0, female C57BL/6J mice were anaesthetized with isoflurane and were orthotopically inoculated in the fourth inguinal mammary fat pad with 50 µL of cell suspension in PBS containing 1 × 10^6^ EO771 breast carcinoma cells (mycoplasma‐free), which were mixed with Matrigel^®^ growth factor reduced matrix (1:1 ratio), immediately before the injection. When the average volume of tumors reached ≈50–100 mm^3^, mice were randomly divided into 3 treatment groups and a control group (5 mice per group). Mice were intravenously (i.v.) treated with 100 µL/animal of BSA1‐SeNPs at dosages of 1.25, 2.5, and 5 mg Kg^‐1^ every 2 days, in the caudal vein. Control mice received an equal volume of PBS.

### Therapeutic Intervention Study Design of the Combination Treatment of BSA1‐SeNPs and the KRAS_wt_ Nanovaccine

On day 0, female C57BL/6J mice were inoculated as previously described. For intervention therapeutic study evaluating the antitumor efficacy of the combinational treatments of the KRAS_*wt*_ nanovaccine and the BSA1‐SeNPs, once the average volume of tumors reached ≈50–100 mm^3^, mice were randomly divided into a control group and 4 treatment groups (n = 6 animals per group), as reported in Table S7 (Supporting Information). KRAS_*wt*_ nanovaccine were subcutaneously (s.c.) administered to mice via injection proximal to both left and right sides inguinal lymph nodes (50 µL per side containing 40 µg of MHC II‐restricted KRAS_*wt*_ peptide antigen, 20 µg of CpG‐ODN and 40 µg of Poly(I:C)), on days 7 and 14 following tumor inoculation. BSA1‐SeNPs were both intratumorally (i.t.) or i.v. administered at 1.25 mg kg^−1^ every 2 days.

### Flow Cytometry Analysis of Immune Subsets

Tumors and spleens were collected from mice (n = 6 animals per group) after euthanasia and homogenized in a single‐cell suspension in cold sterile PBS. Tumor single‐cell suspensions were obtained by mechanical disruption and enzymatic digestion (0.5% (m/v) BSA, 0.1% (m/v) collagenase type II, 0.1% (m/v) neutral protease (dispase) and powders of DNAse I in incomplete RPMI medium) of the tumor tissues, for 30 minutes at 37 °C. After digestion, tumor single‐cell suspensions were filtered through a 70 µm cell strainer with cold PBS to remove the debris. Spleens were also mechanically disrupted, and single‐cell suspensions were depleted of erythrocytes using ACK lysing buffer for 5 minutes at 37 °C, being further filtered, as previously described. Cells were seeded in 96‐well plates, washed with PBS, and incubated with Ghost Dye Red 780 (Cytek Biosciences, Cat# 13‐0865‐T500, 1:5000 for spleen samples and 1:7000 for tumor samples) for 20 minuntes. Cells were then centrifuged (300 *g*, 5 minutes, room temperature) and incubated with FcR Blocking TruStain FcX PLUS CD16/32 (Biolegend, Cat# 156604). After 10 minutes, cells were washed followed by surface staining with extracellular fluorochrome‐labeled anti‐mouse antibodies (listed below), according to the manufacturer's instructions. Cells were then fixed and permeabilized using the eBioscience Foxp3/Transcription Factor Staining Buffer Set (ThermoFisher Scientific, Cat.# 00‐5523‐00) following intracellular staining with fluorochrome‐labeled anti‐mouse antibodies, according to manufacturer's instructions. Samples were analyzed using a a Cytek Aurora cytometer (Cytek) and FlowJo software version 7.6.5 for Microsoft (TreeStar).

### Fluorochrome‐Labeled Anti‐Mouse Antibodies

FoxP3‐eFluor 450 (eBioscience, Cat.# 48‐5773‐82, clone: FJK‐16s, 1:300), NK1.1‐BrilliantViolet (BV)570 (BioLegend, Cat.#108733, clone PK136, 1:20), PD‐1‐Super Bright 600 (Invitrogen, Cat.# 63‐9985‐82, clone J43), CD19‐BV650 (BioLegend, Cat.# 115541, clone 6D5, 1:40), CD3‐FITC (BioLegend, Cat.# 100204, clone: 17A2, 1:80), CD45‐ AlexaFluor (AF)532 (Invitrogen, Cat.# 58‐0451‐82, clone: 30‐F11, 1:80); CD4‐PerCP‐Cy5.5 (BioLegend, Cat.# 100434, clone: GK1.5, 1:80), CD25‐PE/Cyanine5. (BioLegend, Cat.# 102010, clone: PC61, 1:80), CD44‐PE/Cyanine7 (BioLegend, Cat.# 103030, clone: IM7, 1:100), CD8a‐AF700 (BioLegend, Cat.# 100730, clone: 53–6.7, 1:50).

### Haemolysis Assay

The SeNP haemolytic activity was assessed using EDTA‐preserved peripheral mice blood. Plasma was initially removed by centrifugation at 1000 *g* for 10 minutes. Erythrocyte suspension was washed three times with PBS and centrifuged. BSA1‐SeNPs (100 µL) were distributed in 96‐well plates (*n* = 3) at different concentrations ranging from 50 to 400 µg mL^−1^. Erythrocyte suspension (100 µL) was incubated with BSA1‐SeNPs, at 37 °C for 1 hour. Plates were finally centrifuged at 800 *g* for 10 minutes. The absorbance of supernatants was measured at 550 nm using the Varioskan plate reader. The percentage of the haemolytic activity for each sample was calculated by comparing each individual determination to a positive control (erythrocytes incubated with distilled water) corresponding to 100% haemolysis and negative control (erythrocytes incubated with PBS), according to the following equation:

(1) $$\mathrm{Haemolysis}\;\left(\%\right)=\frac{AbsS-AbsN}{AbsP-AbsN}$$

where AbsS is the average absorbance of the sample, AbsN is the average absorbance of the negative control and AbsP is the average absorbance of the positive control.

### Immunohistochemical Analysis

Tumors were recovered post‐animal euthanasia. Tissues were fixed immediately in a 4% buffered formaldehyde solution for 24 hours at 4 °C to proceed to a gelatin embedding and isopentane‐liquid nitrogen freezing method. Samples were stored at −80 °C until sectioning. Gelatine of the 10 µm sections was removed with PBS at 37 °C for 15 minutes. They underwent antigen retrieval by heat (PT link pre‐treatment module for tissue specimens – Thermoscientific at pH 6, Leica Biosystems). Endogenous peroxidase was blocked by incubation with 3% H_2_O_2_ in methanol and a total protein block was also applied (Dako, Cat.# X0909). After the primary antibody anti‐mouse CD8a (1:50; 4SM15; ThermoFisher Scientific, Cat.# 14‐0808‐82) was incubated for 1 hour at room temperature, the ImmPRESS horseradish peroxidase (HRP) goat anti‐rat IgG mouse adsorbed polymer detection kit‐peroxidase was used as a secondary antibody, for 30 minutes at room temperature (Vector Laboratories). Brown positive cells were revealed by enzymatic substrate with HRP DAB solution (containing diaminobenzadine‐tetrahydrochloride; Dako, Cat.# K346811‐2). All slides were counterstained with Harris’ Hematoxylin (bio‐optica) and mounted with Entellan medium (Sigma‐Aldrich, Cat.# 1.00869.0500).

### Biochemical Analysis

Blood was collected by cardiac puncture and centrifuged at 13,000 rpm for 20 minutes at 4 °C to obtain the serum. Serum was delivered to DNAtech (Portugal) to be analyzed. A serum biochemical study evaluated the activity of ALT, GGT, and alkaline phosphatase, known as liver function markers. Creatinine level in serum was also assessed as a marker of kidney function. The basal total proteins and albumin levels were also achieved to support the analysis of both liver and kidney functions. Cardiovascular function was assessed through the serum total cholesterol levels.

### Histopathological Analysis

The major organs (heart, liver, kidney, and spleen) were recovered post‐animal euthanasia. Tissues were fixed in 4% buffered formaldehyde solution for 24 hours at 4 °C, processed overnight using a Tissue HistoCore Pearl (Leica), and embedded in paraffin (Cat.# 39602012, Leica). Paraffin blocks were sectioned into slides, each one with two Sections 3 µm thickness, using a microtome (Minot Microtome Leica RM2145). Slides were stained with hematoxylin (Cat.# 0506004E. Biooptica) and eosin (Cat.# 110132‐1L, Sigma‐Aldrich) (H&E) for morphological examination and histopathological analysis (Instituto Gulbenkian Ciência).

### Statistical Analysis

Data were presented as mean ± standard deviation (s.d.) for stability and in vitro, and mean ± standard error of the mean (s.e.m.) for in vivo assays. Statistical significance was assessed by the Student's *t*‐test, one‐way and two‐way ANOVA, followed by Bonferroni and Tukey multiple comparisons post‐hoc tests for multiple comparisons, using GraphPadPrism 10 (GraphPad Software, Inc.). *p* < 0.05 were considered statistically significant.

### Ethics Approval Statement

All animal procedures were completed in compliance with the Faculty of Pharmacy, University of Lisbon guidelines, and protocols were reviewed and approved by the Direção‐Geral de Alimentação e Veterinária (DGAV, Portugal). Animals were housed under a 12‐hour light, 12‐hour dark cycle, with food and water available *ad libitum* and handled in compliance with the National Institutes of Health (NIH) guidelines and the European Union (EU) rules for the care and handling of laboratory animals (Directive 2010∖63∖EU).

## Conflict of Interest

The authors declare no conflict of interest.

## Supporting information

Supporting Information

## Data Availability

The data that support the findings of this study are available in the supplementary material of this article.

## References

[1] J. Ferlay , M. Colombet , I. Soerjomataram , D. M. Parkin , M. Piñeros , A. Znaor , F. Bray , Int. Agency Res. Cancer 2024, 149, 778.

[2] M. Arnold , E. Morgan , H. Rumgay , A. Mafra , D. Singh , M. Laversanne , J. Vignat , J. R. Gralow , F. Cardoso , S. Siesling , I. Soerjomataram , Breast 2022, 66, 15. 36084384 10.1016/j.breast.2022.08.010 PMC9465273

[3] G. Patel , A. Prince , M. Harries , Semin. Oncol. Nurs. 2024, 40, 151548. 38008654 10.1016/j.soncn.2023.151548

[4] Z. H. Li , P. H. Hu , J. H. Tu , N. S. Yu , Oncotarget 2016, 7, 65024. 27542253 10.18632/oncotarget.11344 PMC5323135

[5] A. Le Naour , Y. Koffi , M. Diab , D. Le Guennec , S. Rougé , S. Aldekwer , N. Goncalves‐Mendes , J. Talvas , M.‐C. Farges , F. Caldefie‐Chezet , M.‐P. Vasson , A. Rossary , Cancer Cell Int. 2020, 20, 328. 32699527 10.1186/s12935-020-01418-1 PMC7372867

[6] S. Zahnreich , H. Schmidberger , Cancers 2021, 13, 2607. 34073340 10.3390/cancers13112607 PMC8198981

[7] Y. Liu , Y. Hu , J. Xue , J. Li , J. Yi , J. Bu , Z. Zhang , P. Qiu , X. Gu , Mol. Cancer 2023, 22, 145. 37660039 10.1186/s12943-023-01850-7 PMC10474743

[8] M. Rasool , A. Malik , S. Waquar , M. Arooj , S. Zahid , M. Asif , S. Shaheen , A. Hussain , H. Ullah , S. Hua , Bioengineered 2021, 13, 759. 10.1080/21655979.2021.2012907 PMC8805951 34856849

[9] S. Jahan , M. E. Karim , E. H. Chowdhury , Biomedicines 2021, 9, 114. 33530291 10.3390/biomedicines9020114 PMC7910939

[10] M. A. Rahim , N. Jan , S. Khan , H. Shah , A. Madni , A. Khan , A. Jabar , S. Khan , A. Elhissi , Z. Hussain , H. C. Aziz , M. Sohail , M. Khan , H. E. Thu , Cancers (Basel), 2021, 13, 670. 10.3390/cancers13040670 PMC7914759 33562376

[11] L. Palanikumar , S. Al‐hosani , M. Kalmouni , V. P. Nguyen , F. N. Barrera , M. Magzoub , Commun. Biol. 2020, 3, 95. 32127636 10.1038/s42003-020-0817-4 PMC7054360

[12] H. Wu , H. Zhuzhu , X. Li , Z. Li , W. Zheng , T. Chen , B. Yu , K. H. Wong , J. Agric. Food Chem. 2013, 61, 9859. 24053442 10.1021/jf403564s

[13] A. A. Abd‐Rabou , H. H. Ahmed , A. B. Shalby , Biol. Trace Elem. Res. 2020, 193, 377. 31066020 10.1007/s12011-019-01730-6

[14] A. P. Fernandes , V. Gandin , Biochim. Biophys. Acta – Gen. Subj. 2015, 1850, 1642. 10.1016/j.bbagen.2014.10.008 25459512

[15] S. Hariharan , S. Dharmaraj , Inflammopharmacology 2020, 28, 667. 32144521 10.1007/s10787-020-00690-x PMC7222958

[16] C. Ferro , H. F. Florindo , H. A. Santos , Adv. Healthcare Mater. 2021, 10, 2100598. 10.1002/adhm.202100598 34121366

[17] D. Cui , C. Yan , J. Miao , X. Zhang , J. Chen , L. Sun , L. Meng , T. Liang , Q. Li , Mater. Sci. Eng. C 2018, 90, 104. 10.1016/j.msec.2018.04.048 29853073

[18] G. Zhao , X. Wu , P. Chen , L. Zhang , C. S. Yang , J. Zhang , Biol. Med. 2018, 126, 55. 10.1016/j.freeradbiomed.2018.07.017 30056082

[19] G. Chen , F. Yang , S. Fan , H. Jin , K. Liao , X. Li , G. Bin Liu , J. Liang , J. Zhang , J. F. Xu , J. Pi , Front. Immunol. 2022, 13, 956181. 35958612 10.3389/fimmu.2022.956181 PMC9361286

[20] K. Bai , B. Hong , J. He , Z. Hong , R. Tan , Int. J. Nanomed. 2017, 12, 4527. 10.2147/IJN.S129958 PMC5485894 28684913

[21] X. Zhang , H. Yan , L. Ma , H. Zhang , D. F. Ren , J. Food Biochem. 2020, 44, e13363. 32648615 10.1111/jfbc.13363

[22] S. Menon , S. D. Ks , S. R , R. S , V. K. S , Colloids Surf. B Biointerfaces 2018, 170, 280. 29936381 10.1016/j.colsurfb.2018.06.006

[23] W. Chen , L. Yue , Q. Jiang , W. Xia , IET Nanobiotechnol. 2019, 13, 30. 30964034 10.1049/iet-nbt.2018.5052 PMC8676009

[24] S. Chung , R. Zhou , T. J. Webster , Int. J. Nanomed. 2020, 15, 115. 10.2147/IJN.S193886 PMC6955603 32021168

[25] A. Selmani , L. Ulm , K. Kasemets , I. Kurvet , I. Erceg , R. Barbir , B. Pem , P. Santini , I. D. Marion , T. Vinkovic , A. Krivohlavek , M. D. Sikiric , A. Kahru , I. Vinkovic Vrcek , Chemosphere 2020, 250, 126265. 32109702 10.1016/j.chemosphere.2020.126265

[26] G. Khiralla , H. Elhariry , J. Food Biochem. 2020, 44, e13413. 32748421 10.1111/jfbc.13413

[27] A. A.‐R. Mohamed , S. I. Khater , A. H. Arisha , M. M. M. Metwally , G. Mostafa‐Hedeab , E. S. El‐Shetry , Gene 2021, 768, 145288. 33181259 10.1016/j.gene.2020.145288

[28] S. Zhao , Y. Luo , Z. Chang , C. Liu , T. Li , L. Gan , Y. Huang , Q. Sun , Nanoscale Res. Lett. 2021, 16, 170. 34842995 10.1186/s11671-021-03627-7 PMC8630206

[29] X. Shen , X. Liu , T. Li , Y. Chen , Y. Chen , P. Wang , L. Zheng , H. Yang , C. Wu , S. Deng , Y. Liu , Front. Chem. 2021, 9, 746646. 34869202 10.3389/fchem.2021.746646 PMC8636905

[30] F. Maiyo , M. Singh , Biomedicines 2020, 8, 76. 32260507 10.3390/biomedicines8040076 PMC7235796

[31] X. Song , Y. Chen , G. Zhao , H. Sun , H. Che , X. Leng , Carbohydr. Polym. 2020, 231, 115689. 31888818 10.1016/j.carbpol.2019.115689

[32] M. Shahbazi , P. V. Almeida , E. Mäkilä , A. Correia , M. P. a Ferreira , M. Kaasalainen , J. Salonen , J. Hirvonen , H. a Santos , Macromol. Rapid Commun. 2014, 35, 624. 24497275 10.1002/marc.201300868

[33] H. Zhan , F. Ma , Y. Huang , J. Zhang , X. Jiang , Y. Qian , Eur. J. Pharm. Sci. 2018, 121, 330. 29908904 10.1016/j.ejps.2018.06.014

[34] M. A. Shahbazi , P. V. Almeida , A. Correia , B. Herranz‐Blanco , N. Shrestha , E. Mäkilä , J. Salonen , J. Hirvonen , H. A. Santos , J. Controlled Release 2017, 249, 111. 10.1016/j.jconrel.2017.01.046 28159519

[35] R. Ganassin , F. H. Horst , N. S. Camargo , S. B. Chaves , P. C. Morais , E. Mosiniewicz‐Szablewska , P. Suchocki , J. P. Figueiró Longo , R. B. Azevedo , L. A. Muehlmann , Artif. Cells, Nanomed. Biotechnol 2018, 46, 1046. 29842818 10.1080/21691401.2018.1478423

[36] J. Gao , J. Liu , F. Xie , Y. Lu , C. Yin , X. Shen , Int. J. Nanomed. 2019, 14, 9199. 10.2147/IJN.S230376 PMC6884979 32063706

[37] M. K. Viswanadh , N. Agrawal , S. Azad , A. Jha , S. Poddar , S. K. Mahto , M. S. Muthu , Int. J. Pharm. 2021, 602,. 10.1016/j.ijpharm.2021.120652 33915187

[38] M. Danaei , M. Dehghankhold , S. Ataei , F. Hasanzadeh Davarani , R. Javanmard , A. Dokhani , S. Khorasani , M. R. Mozafari , Pharmaceutics 2018, 10, 57. 29783687 10.3390/pharmaceutics10020057 PMC6027495

[39] N. Raval , R. Maheshwari , D. Kalyane , S. R. Youngren‐Ortiz , M. B. Chougule , R. K. Tekade , Importance of Physicochemical Characterization of Nanoparticles in Pharmaceutical Product Development, Elsevier Inc., New York 2019.

[40] S. Zeng , Y. Ke , Y. Liu , Y. Shen , L. Zhang , C. Li , A. Liu , L. Shen , X. Hu , H. Wu , W. Wu , Y. Liu , Colloids Surf., B 2018, 170, 115. 10.1016/j.colsurfb.2018.06.003 29894831

[41] T. G. F. Souza , V. S. T. Ciminelli , N. D. S. Mohallem , J. Phys. Conf. Ser. 2016, 733, 012039.

[42] M. Sharifiaghdam , E. Shaabani , Z. Sharifiaghdam , H. De Keersmaecker , R. De Rycke , S. De Smedt , R. Faridi‐Majidi , K. Braeckmans , J. C. Fraire , Front. Mol. Biosci. 2021, 8, 639184. 33959633 10.3389/fmolb.2021.639184 PMC8093573

[43] S. Pandey , N. Awasthee , A. Shekher , L. C. Rai , S. C. Gupta , S. K. Dubey , Bioprocess Biosyst. Eng. 2021, 44, 2679. 34599397 10.1007/s00449-021-02637-0

[44] E. S. Bronze‐Uhle , B. C. Costa , V. F. Ximenes , P. N. Lisboa‐Filho , Nanotechnol. Sci. Appl. 2017, 10, 11. 28096662 10.2147/NSA.S117018 PMC5207451

[45] K. Nawrotek , M. Tylman , K. Rudnicka , J. Balcerzak , Carbohydr. Polym. 2016, 136, 764. 26572411 10.1016/j.carbpol.2015.09.105

[46] M. F. Queiroz , K. R. T. Melo , D. A. Sabry , G. L. Sassaki , H. A. O. Rocha , Mar. Drugs 2015, 13, 141. 10.3390/md13010141 PMC4306929 25551781

[47] Y. K. Demir , A. Ü. Metin , B. Şatıroğlu , M. E. Solmaz , V. Kayser , K. Mäder , Eur. J. Pharm. Biopharm. 2017, 117, 182. 28438549 10.1016/j.ejpb.2017.04.018

[48] Y. Zheng , H. Chen , X. Zeng , Z. Liu , X. Xiao , Y. Zhu , D. Gu , L. Mei , Nanoscale Res. Lett. 2013, 8, 1. 23570619 10.1186/1556-276X-8-161 PMC3639870

[49] SigmaAldrich, “IR Spectrum Table & Chart,” can be found under https://www.sigmaaldrich.com/technical‐documents/articles/biology/ir‐spectrum‐table.html, (accessed: May 2021).

[50] T. Topală , A. Bodoki , L. Oprean , R. Oprean , Clujul Med. 2014, 87, 5. 10.15386/cjmed-357 PMC4620676 26528027

[51] M. J. Ye , Q. L. Xu , H. Y. Tang , W. Y. Jiang , D. X. Su , S. He , Q. Z. Zeng , Y. Yuan , LWT 2020, 126, 109280.

[52] E. Trenkenschuh , W. Friess , Eur. J. Pharm. Biopharm. 2021, 165, 345. 34052428 10.1016/j.ejpb.2021.05.024

[53] X. Song , Y. Chen , H. Sun , X. Liu , X. Leng , Food Chem. 2020, 331, 127378. 32593797 10.1016/j.foodchem.2020.127378

[54] D. Chen , S. Ganesh , W. Wang , M. Amiji , AAPS J. 2020, 22, 1. 10.1208/s12248-020-00464-x 32495039

[55] F. Alexis , E. Pridgen , L. K. Molnar , O. C. Farokhzad , Mol. Pharmaceutics 2008, 5, 505. 10.1021/mp800051m PMC2663893 18672949

[56] T. L. Moore , L. Rodriguez‐lorenzo , V. Hirsch , S. Balog , D. Urban , C. Jud , B. Rothen‐rutishauser , A. Petri‐fink , Chem. Soc. Rev. 2015, 44, 6287. 26056687 10.1039/c4cs00487f

[57] A. R. Shahverdi , F. Shahverdi , E. Faghfuri , M. Reza khoshayand , F. Mavandadnejad , M. H. Yazdi , M. Amini , Arch. Med. Res. 2018, 49, 10. 29699810 10.1016/j.arcmed.2018.04.007

[58] E. Faghfuri , R. Ajideh , F. Shahverdi , M. Hosseini , F. Mavandadnejad , M. H. Yazdi , A. R. Shahverdi , Avicenna J. Med. Biotechnol. 2021, 13, 201. 34900146 PMC8606114

[59] A. A. Abd‐Rabou , A. B. Shalby , H. H. Ahmed , Biol. Trace Elem. Res. 2019, 187, 80. 29748931 10.1007/s12011-018-1360-8

[60] S. H. Jalalian , M. Ramezani , K. Abnous , S. M. Taghdisi , Cancer Lett. 2018, 416, 87. 29253524 10.1016/j.canlet.2017.12.023

[61] S. Gao , T. Li , Y. Guo , C. Sun , B. Xianyu , H. Xu , Adv. Mater. 2020, 32, 1907568. 10.1002/adma.201907568 32053267

[62] J. Zou , S. Su , Z. Chen , F. Liang , Y. Zeng , W. Cen , X. Zhang , Y. Xia , D. Huang , Artif. Cells, Nanomed. Biotechnol. 2019, 47, 3456. 31469318 10.1080/21691401.2019.1626863

[63] Y. Xia , Y. Chen , L. Hua , M. Zhao , T. Xu , C. Wang , Y. Li , B. Zhu , Int. J. Nanomed. 2018, 13, 6929. 10.2147/IJN.S174909 PMC6214589 30464451

[64] A. P. Bidkar , P. Sanpui , S. S. Ghosh , Nanomedicine 2017, 12, 2641. 29043926 10.2217/nnm-2017-0189

[65] D. B. Keskin , A. J. Anandappa , J. Sun , I. Tirosh , N. D. Mathewson , S. Li , G. Oliveira , A. Giobbie‐Hurder , K. Felt , E. Gjini , S. A. Shukla , Z. Hu , L. Li , P. M. Le , R. L. Allesøe , A. R. Richman , M. S. Kowalczyk , S. Abdelrahman , J. E. Geduldig , S. Charbonneau , K. Pelton , J. B. Iorgulescu , L. Elagina , W. Zhang , O. Olive , C. McCluskey , L. R. Olsen , J. Stevens , W. J. Lane , A. M. Salazar , et al., Nature 2019, 565, 234. 30568305 10.1038/s41586-018-0792-9 PMC6546179

[66] N. Hilf , S. Kuttruff‐Coqui , K. Frenzel , V. Bukur , S. Stevanovic , C. Gouttefangeas , M. Platten , G. Tabatabai , V. Dutoit , S. H. van der Burg , P. thor Straten , F. Martínez‐Ricarte , B. Ponsati , H. Okada , U. Lassen , A. Admon , C. H. Ottensmeier , A. Ulges , S. Kreiter , A. von Deimling , M. Skardelly , D. Migliorini , J. R. Kroep , M. Idorn , J. Rodon , J. Piró , H. S. Poulsen , B. Shraibman , K. McCann , R. Mendrzyk , et al., Nature 2019, 565, 240. 30568303 10.1038/s41586-018-0810-y

[67] Z. Hu , D. E. Leet , R. L. Allesøe , G. Oliveira , S. Li , A. M. Luoma , J. Liu , J. Forman , T. Huang , J. B. Iorgulescu , R. Holden , S. Sarkizova , S. H. Gohil , R. A. Redd , J. Sun , L. Elagina , A. Giobbie‐Hurder , W. Zhang , L. Peter , Z. Ciantra , S. Rodig , O. Olive , K. Shetty , J. Pyrdol , M. Uduman , P. C. Lee , P. Bachireddy , E. I. Buchbinder , C. H. Yoon , D. Neuberg , et al., Nat. Med. 2021, 27, 515. 33479501 10.1038/s41591-020-01206-4 PMC8273876

[68] L. M. Mustachio , A. Chelariu‐Raicu , L. Szekvolgyi , J. Roszik , Cancers 2021, 13, 1204. 33801965 10.3390/cancers13061204 PMC7999304

[69] E. Zhang , C. Ding , S. Li , X. Zhou , B. Aikemu , X. Fan , J. Sun , M. Zheng , X. Yang , Biomark. Res. 2023, 11, 28. 36890557 10.1186/s40364-023-00460-1 PMC9997025

[70] Y. Togashi , K. Shitara , H. Nishikawa , Nat. Rev. Clin. Oncol. 2019, 16, 356. 30705439 10.1038/s41571-019-0175-7

[71] S. Pan , T. Li , Y. Tan , H. Xu , Biomaterials 2022, 280, 121321. 34922271 10.1016/j.biomaterials.2021.121321

[72] H. F. Florindo , S. Pandit , L. M. D. Gonçalves , M. Videira , O. Alpar , A. J. Almeida , Biomaterials 2009, 30, 5161. 19524290 10.1016/j.biomaterials.2009.05.045

[73] I. V. Mikheev , E. A. Karpukhina , L. O. Usol'tseva , T. O. Samarina , D. S. Volkov , M. A. Proskurnin , Inorg. Mater. 2017, 53, 1422.

[74] Y. Liu , L. Wang , J. Liu , X. Xie , H. Hu , F. Luo , Cancer Manag. Res. 2020, 12, 5131. 32617021 10.2147/CMAR.S244748 PMC7326172

[75] V. L. Silva , D. Ferreira , F. L. Nobrega , I. M. Martins , L. D. Kluskens , L. R. Rodrigues , PLoS One 2016, 11, e0161290. 27548261 10.1371/journal.pone.0161290 PMC4993384

[76] H. Eguchi , R. Akizuki , R. Maruhashi , M. Tsukimoto , T. Furuta , T. Matsunaga , S. Endo , A. Ikari , Sci. Rep. 2018, 8, 15157. 30310131 10.1038/s41598-018-33566-w PMC6181945

[77] P. Figueiredo , C. Ferro , M. Kemell , Z. Liu , A. Kiriazis , K. Lintinen , H. F. Florindo , J. Yli‐Kauhaluoma , J. Hirvonen , M. A. Kostiainen , H. A. Santos , Nanomedicine 2017, 12, 2581. 28960138 10.2217/nnm-2017-0219

