# Supplementary material for: Selenium Nanoparticles Synergize with a KRAS Nanovaccine against Breast Cancer
Source: Adv Healthc Mater. 2024 Aug 29;14(5):2401523. doi: 10.1002/adhm.202401523 (PMC11834378; doi:10.1002/adhm.202401523)
Supplement: Supplementary file 1 — Supporting Information [file ADHM-14-0-s001.docx]

Supporting Information

Selenium nanoparticles synergize with a KRAS nanovaccine against breast cancer

Cláudio Ferro^1,2^, Ana I. Matos^1^, Luigia Serpico^3^, Flavia Fontana^2^, Jacopo Chiaro^4^, Carmine D'Amico^2^, Alexandra Correia^2^, Risto Koivula^5^, Marianna Kemell^5^, Maria Manuela Gaspar^1^, Rita C. Acúrcio^1^, Vincenzo Cerullo^3,4^, Hélder A. Santos^2,6*^, Helena F. Florindo^1*^

**Table S1.** Determination of the Se concentration (ppm), the number of SeNP, and the amount of Se within each type of SeNP: BSA1-SeNPs; BSA12-SeNPs; Chitosan-SeNPs; PMVEMA-SeNPs and TPGS-SeNPs.

|  | BSA1-SeNPs | BSA12-SeNPs | Chitosan-SeNPs | PMVEMA-SeNPs | TPGS-SeNPs |
| --- | --- | --- | --- | --- | --- |
| Se quantification (ppm) | 1001.3 ± 30.0 | 274.0 ± 8.0 | 370.0 ± 11.0 | 724.3 ± 22 | 516.0 ± 15.9 |
| Particle's concentration (particles mL^-1^) | 4.1x10^12^ ± 2.6x10^11^ | 2.1x10^12^ ± 4.4x10^11^ | 3.8x10^12^ ± 2.4x10^11^ | 1.8x10^12^ ± 3.6x10^11^ | 6.8x10^12^ ± 3.3x10^11^ |
| Amount of Se per particle (mg particle^-1^) | 2.4x10^-13^ | 1.3x10^-13^ | 9.7x10^-14^ | 4.0x10^-13^ | 7.5x10^-14^ |

BSA: bovine serum albumin; PMVEMA: polymethyl vinyl ether-alt-maleic anhydride; Se: selenium; SeNP: selenium nanoparticles; TPGS: tocopherol polyethylene glycol succinate.

**Table S2.** Elemental analysis results for each type of SeNP produced: BSA1-SeNP; BSA12-SeNP; Chitosan-SeNP; PMVEMA-SeNP and TPGS-SeNPs.

|  | N (%) | C (%) | H (%) | S (%) | Total (%) |
| --- | --- | --- | --- | --- | --- |
| **BSA1-SeNPs** | 7.78 ± 0.14 | 26.78 ± 0.62 | 3.88 ± 0.08 | 1.10 ± 0.22 | 39.54 ± 1.06 |
| **BSA12-SeNPs** | 9. 90 ± 0.09 | 36.08 ± 0.14 | 5.18 ± 0.06 | 1.03 ± 0.07 | 52.19 ± 0.36 |
| **Chitosan-SeNPs** | 3.46 ± 0.03 | 23.09 ± 0.31 | 4.17 ± 0.09 | 0.05 ± 0.03 | 30.77 ± 0.46 |
| **PMVEMA-SeNPs** | 0.05 ± 0.01 | 8.82 ± 0.31 | 1.32 ± 0.04 | 0.03 ± 0.02 | 10.22 ± 0.38 |
| **TPGS-SeNPs** | 0.05 ± 0.01 | 9.53 ± 0.34 | 1.54 ± 0.05 | 0.07 ± 0.01 | 11.19 ± 0.41 |

BSA: bovine serum albumin; PMVEMA: polymethyl vinyl ether-alt-maleic anhydride; SeNP: selenium nanoparticles; TPGS: tocopherol polyethylene glycol succinate.

| Time point | SeNP | Cell lines | | |
| --- | --- | --- | --- | --- |
|  |  | 4T1 | A549 | Fibroblasts |
| 24 hours | BSA1-SeNPs | *2 | 1233.5 ± 381.3 | 1111.6 ± 118.8 |
|  | BSA12-SeNPs | 534.7 ± 38.8 | 364.6 ± 25.5 | 2604.1 ± 193.7 |
|  | Chitosan-SeNPs | 69.8 ± 0.8 | 58.3 ± 2.3 | 344.9 ± 21.5 |
|  | PMVEMA-SeNPs | 410.1 ± 31.5 | 460.1 ± 25.3 | 645.9 ± 139.3 |
|  | TPGS-SeNPs | 36.3 ± 25.4 | 35.0 ± 18.6 | 203.9 ± 17.9 |
|  | Sodium Selenite | *1 | *1 | 657.3 ± 213.2 |
| 48 hours | BSA1-SeNPs | 113.8 ± 1.4 | 19.9 ± 5.0 | 836.5 ± 62.7 |
|  | BSA12-SeNPs | 122.0 ± 1.0 | *1 | *2 |
|  | Chitosan-SeNPs | *1 | *1 | 385.4 ± 13.8 |
|  | PMVEMA-SeNPs | 48.0 ± 2.8 | 15.7 ± 10.6 | 1070.7 ± 258.2 |
|  | TPGS-SeNPs | 42.0 ± 1.9 | 30.5 ± 5.4 | 51.4 ± 10.7 |
|  | Sodium Selenite | *1 | *1 | *1 |

**Table S3.** IC_50_ values of each type of SeNP: BSA1-SeNP; BSA12-SeNP; Chitosan-SeNP; PMVEMA-SeNP and TPGS-SeNP. The IC_50_ values were determined at µg mL^-1^ for different cell lines and the incubation time points of 24 and 48 hours.

*1 – SeNP antiproliferative effect below 10 µg mL^-1^.

*2 - SeNP antiproliferative effect above 3000 µg mL^-1^.

BSA: Bovine Serum Albumin; SeNP: Selenium Nanoparticles; PMVEMA: Polymethyl vinyl ether-alt-maleic anhydride; TPGS: Tocopherol polyethylene glycol succinate.

**Table S4.** IC_50_ values of pure materials used as SeNP stabilizers: BSA; Chitosan; PMVEMA and TPGS. The IC_50_ values were determined at µg mL^-1^ for different cell lines for 48 hours of incubation.

| Stabilizers | Cell lines | | |
| --- | --- | --- | --- |
|  | 4T1 | EO771 | Fibroblasts |
| BSA | 1701.5 ± 200.3 | *2 | *2 |
| Chitosan | 845.3 ± 113.7 | 958.4 ± 184.8 | 582.3 ± 123.1 |
| PMVEMA | 1590.8 ± 290.2 | 531.5 ± 32.7 | 774.1 ± 187.2 |
| TPGS | 609.0 ± 39.0 | 171.5 ± 25.5 | 320.7 ± 42.2 |

*2 - SeNP antiproliferative effect above 3000 µg mL^-1^.

BSA: Bovine Serum Albumin; SeNP: Selenium Nanoparticles; PMVEMA: Polymethyl vinyl ether-alt-maleic anhydride; TPGS: Tocopherol polyethylene glycol succinate.

**Table S5.** Haemolytic activity (%) of BSA1-SeNPs (*n* = 3) in mouse RBC.

| **SeNP concentration (μg mL^-1^)** | **Haemolytic activity in RBC (%)** |
| --- | --- |
| 400 | 0.66 |
| 200 | 0.36 |
| 100 | 0.01 |
| 50 | 0 |

BSA: bovine serum albumin; RBC: red blood cells; SeNPs: selenium nanoparticles.

**Table S6.** SeNP preparation protocol for elemental analysis.

| Method name | Graphite120s |
| --- | --- |
| O_2_ dosing time | 120 s |
| Autozero delay N | 10 s |
| Autozero delay S | 10 s |
| Peak anticipation N | 40 s |
| Peak anticipation C | 120 s |
| Peak anticipation H | 50 s |
| Peak anticipation S | 70 s |
| Desorption CO_2_ | 60°C |
| Desorption H_2_O | 140 s |
| Desorption CO_2_ | 210°C |
| Temp. of the combustion tube | 1150°C |
| Temp. of the reduction tube | 850°C |
| He flow | ~200 mL minute^-1^ |
| O_2_ flow | 28-30 mL minute^-1^ |

1^st^. Combustion tube (Ø 20 mm) was filled with: Quartz wool, Wolfram (VI) oxide-granulate, Corundum balls, Ash crucible with bottom of Al_2_O_3_ –wool.

2^nd^. Reduction tube (Ø 20 mm) was filled with: Quartz wool, Copper, Corundum balls and Silver wool.

Test substance for elementary analysis: Sulfanilamide, from Elementar, made in Germany.

Tin boats 4x4x11 mm were used to weight samples.

Balance: Mettler Toledo MX5.

TCD-detector.

**Table S7.** Therapeutic groups and administration routes for SeNP combinatorial assays.

| Group | Treatment | Route of administration |
| --- | --- | --- |
| 1 | Control | – |
| 2 | Nanovaccine | s.c. |
| 3 | BSA1-SeNP | i.t. |
| 4 | BSA1-SeNP + Nanovaccine | i.v. + s.c. |
| 5 | BSA1-SeNP + Nanovaccine | i.t. + s.c. |

i.t.: intratumoral; i.v.: intravenous; NP: nanoparticle; s.c.: subcutaneous; SeNP: selenium nanoparticles. Nanovaccine: NP loading MHC II-restricted KRAS*_wt_* peptide antigen and TLR ligands (CpG-ODN, Poly(I:C)).

**Supplementary Figures**


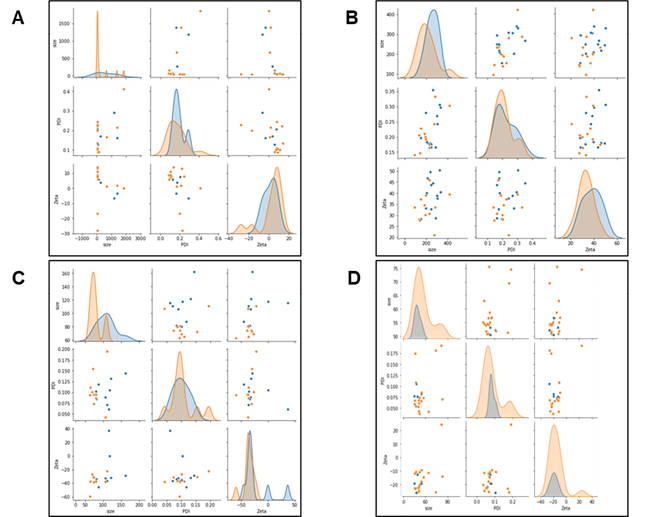


**Figure S1.** Pair plot concerning the relation between the mean average size (nm), PdI and the ζ-potential (mV) and the production method (blue – method 1; orange – method 2 for the different SeNP produced using BSA (A), Chitosan (B), PMVE-MA (C), and TPGS (D) as stabilizers.

BSA: bovine serum albumin; PMVEMA: polymethyl vinyl ether-alt-maleic anhydride; SeNP: selenium nanoparticles; TPGS: tocopherol polyethylene glycol succinate.


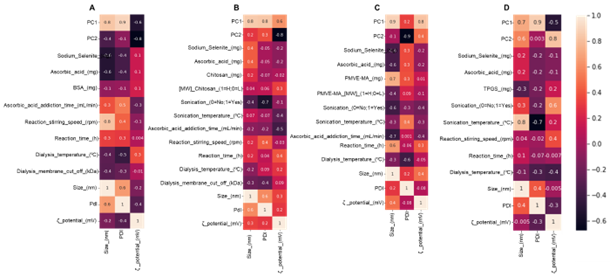


**Figure S2.** Heat-maps displaying the correlation between the several parameters optimized for SeNP production and their size, PDI and ζ-potential: A) BSA-SeNPs; B) Chitosan-SeNPs; C) PMVAME-SeNPs; D) TPGS-SeNPs.

BSA: bovine serum albumin; PMVEMA: polymethyl vinyl ether-alt-maleic anhydride; SeNP: selenium nanoparticles; TPGS: tocopherol polyethylene glycol succinate.


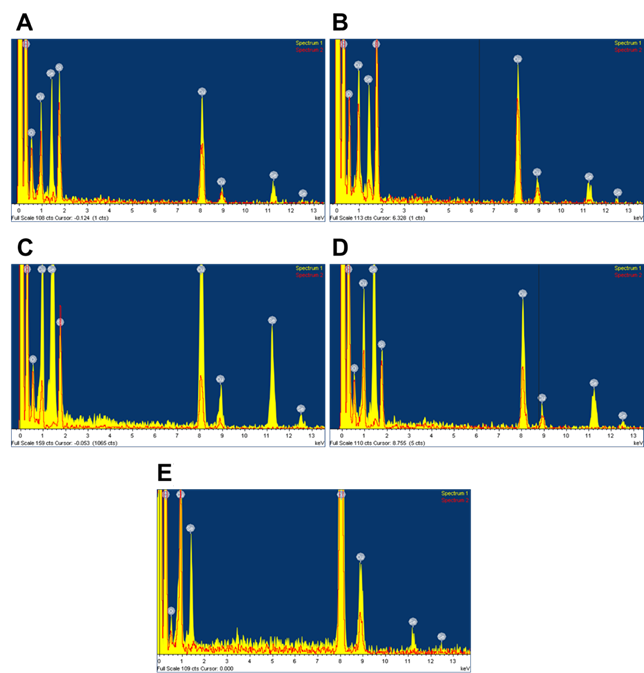


**Figure S3.** Energy-Dispersive X-Ray Spectroscopy (EDS) analysis confirming the presence of Se within the SeNP: BSA1-SeNPs; BSA12-SeNP; Chitosan-SeNPs; PMVEMA-SeNPs and TPGS-SeNPs.

BSA: bovine serum albumin; PMVEMA: polymethyl vinyl ether-alt-maleic anhydride; SeNP: selenium nanoparticles; TPGS: tocopherol polyethylene glycol succinate.


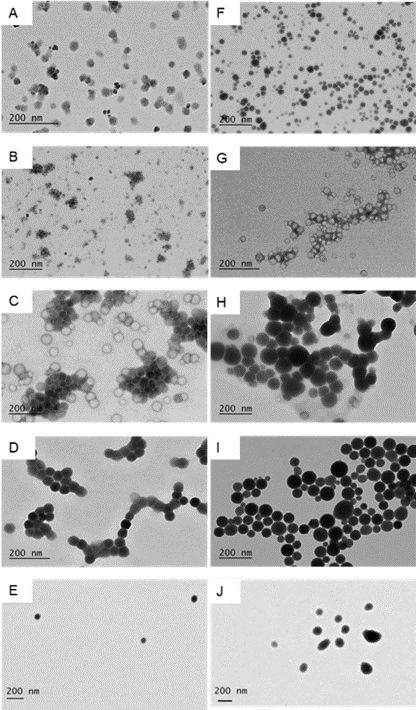


**Figure S4.** Transmission electron microscopy (TEM) images of the different SeNP after 4-week storage at 4ºC (A-E) or at room temperature (the scale bars are presented in the individual pictures): BSA1-SeNPs (A and F); BSA12-SeNP (B and G); Chitosan-SeNPs (C and H); PMVEMA-SeNPs (D and I); TPGS-SeNPs (E and J).

BSA: bovine serum albumin; PMVEMA: polymethyl vinyl ether-alt-maleic anhydride; SeNP: selenium nanoparticles; TPGS: tocopherol polyethylene glycol succinate.


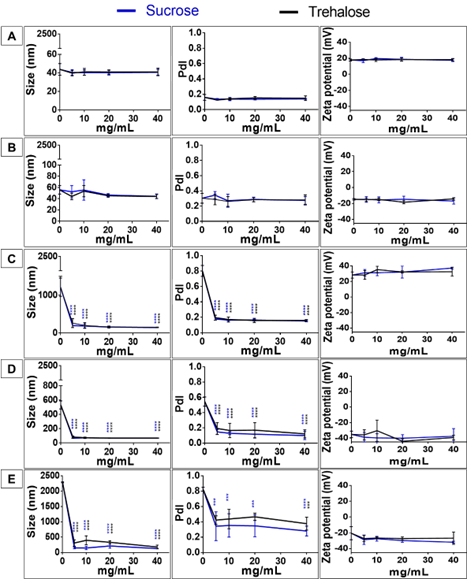


**Figure S5.** Freeze-drying effect on SeNPs physicochemical properties and the influence of sucrose (blue) and trehalose (black), at distinct concentrations, as cryoprotectants: BSA1-SeNPs (A), BSA12-SeNPs (B), Chitosan-SeNPs (C), PMVEMA-SeNPs (D), TPGS-SeNPs (E). Error bars represent the mean ± s.d. (n = 3). Data were analyzed with two-way ANOVA, followed by Bonferroni post-hoc test to evaluate how each cryoprotectant and respective concentrations affect SeNP properties, compared to SeNP freeze-dried without cryoprotectant (grey – trehalose; black – sucrose). The levels of significance were set at the probabilities of ^***^*p* < 0.001 and ^****^*p* < 0.0001.

BSA: bovine serum albumin; PMVEMA: polymethyl vinyl ether-alt-maleic anhydride; SeNP: selenium nanoparticles; TPGS: tocopherol polyethylene glycol succinate.


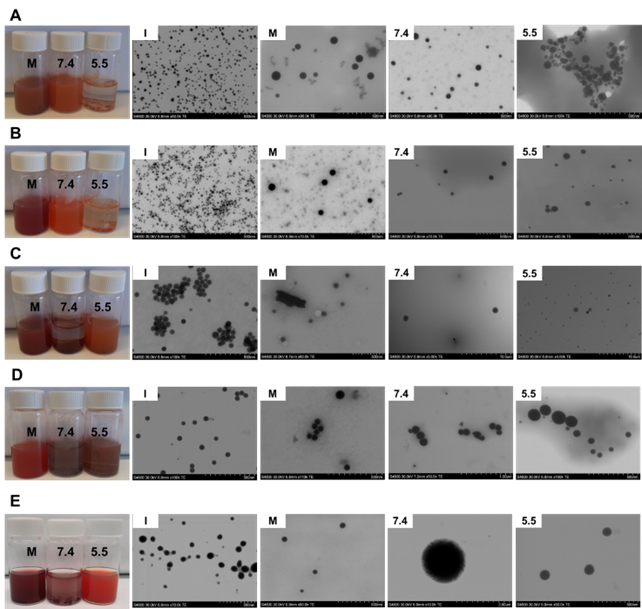


**Figure S6.** SeNP long-term stability after incubation in HEPES pH 7.4, MES pH 5.5, and RPMI with 10% FBS for 1 week. After 1 week of incubation, the samples were photographed. Shape and size of SeNP before (I) and after incubation with cell medium (M), HEPES pH 7.4 (7.4), and MES pH 5.5 (5.5) were evaluated by BF-TE imaging (the scale bars are presented in the individual pictures): BSA1-SeNPs (A); BSA12-SeNP (B); Chitosan-SeNPs (C); PMVEMA-SeNPs (D); TPGS-SeNPs (E).

BSA: bovine serum albumin; PMVEMA: polymethyl vinyl ether-alt-maleic anhydride; SeNP: selenium nanoparticles; TPGS: tocopherol polyethylene glycol succinate.


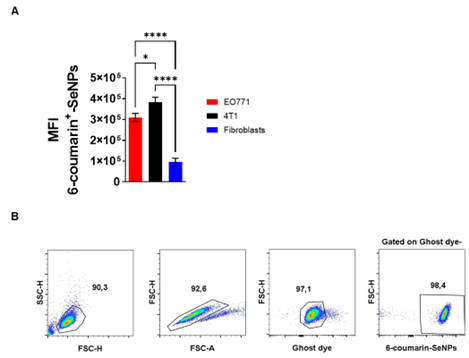


**Figure S7.** Flow cytometry analysis of 6-coumarin^+^-SeNPs internalization (**A**) by murine 4T1 TNBC and EO771 luminal B breast cancer cells, and fibroblasts after incubation of 6-coumarin-labeled BSA1-SeNPs (100 µg mL^-1^), for 3 hours. Cells in complete medium were used as control. Data are presented as mean ± s.d. (n = 3). **p* < 0.05, *****p* < 0.0001, analyzed by one-way ANOVA with Tukey multiple comparisons post-hoc test. (**B**) Representative gating strategy used for 6-coumatin-SeNPs internalization analysis.


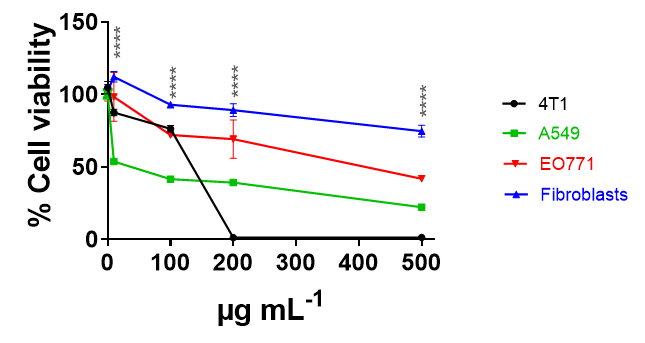


**Figure S8.** Antiproliferative studies of 4T1, EO771, A549 and human fibroblast cells treated with different concentrations (10, 100, 200 and 500 μg mL^-1^) of BSA1-SeNPs prepared in complete medium, at 37^◦^C, for 48 hours. Cells in complete medium were used as control. Data are presented as mean ± s.d. (*n* = 3). Statistical significance between the difference in viability among the cell lines studied for each concentration was analyzed by two-way ANOVA followed by Bonferroni’s post-test. The levels of significance were set at the probabilities of **** *p* < 0.0001.

**
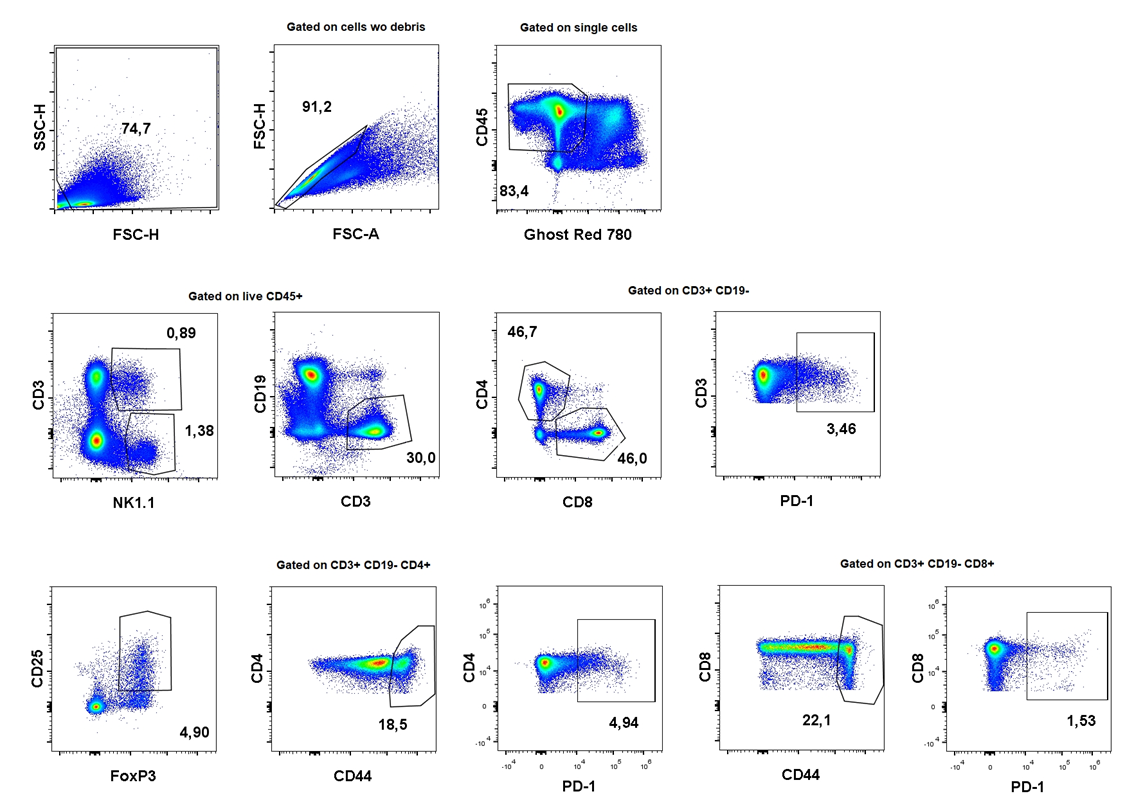
**

**Figure S9.** Representative gating strategy used for the activated and PD1-expressing T lymphocytes, Treg and NK cells in the spleen samples: T (CD45^+^ CD3^+^ CD19^-^), PD1-expressing T (CD45^+^ CD3^+^ CD19^-^ PD1^+^), CD4 T (CD45^+^ CD3^+^ CD19^-^ CD8a^-^ CD4^+^), Treg (CD45^+^ CD3^+^ CD19^-^ CD8a^-^ CD4^+^ CD25^+^ FoxP3^+^), activated CD4 T (CD45^+^ CD3^+^ CD19^-^ CD8a^-^ CD4^+^ CD44^+^), PD1-expressing CD4 T (CD45^+^ CD3^+^ CD19^-^ CD8a^-^ CD4^+^ PD1^+^), activated CD8 T (CD45^+^ CD3^+^ CD19^-^ CD8α^+^ CD4^-^ CD44^+^), PD1-expressing CD8 T (CD45^+^ CD3^+^ CD19^-^ CD8a^+^ CD4^-^ PD1^+^), NKT (CD45^+^ CD3^+^ NK1.1^+^) and NK ( CD45^+^ CD3^-^ NK1.1^+^) cells.


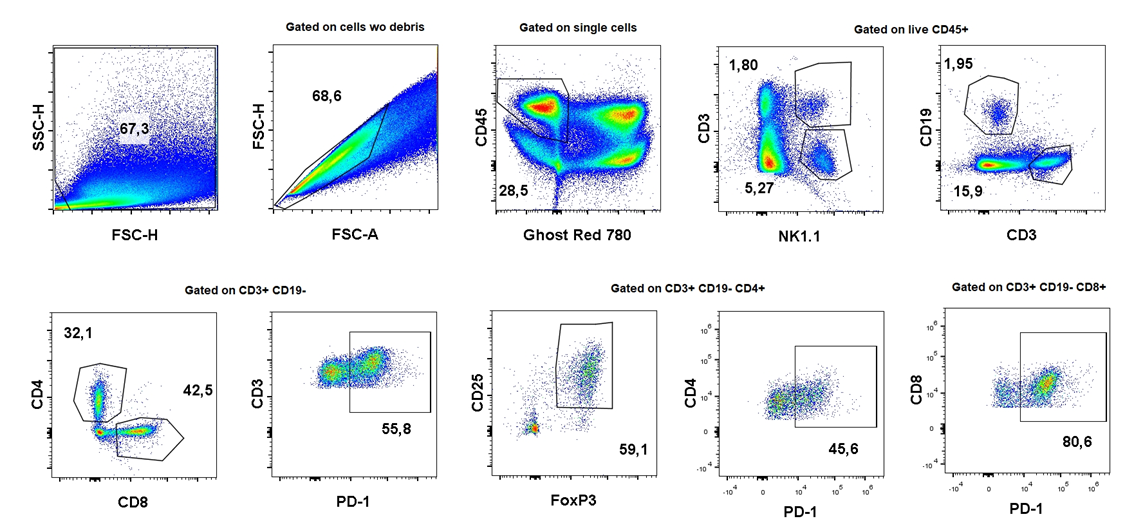


**Figure S10.** Representative gating strategy used for the B and T Lymphocytes, NK and PD1-expressing T cells in the tumor samples: B (CD45^+^ CD3^-^ CD19^+^), T (CD45^+^ CD3^+^ CD19^-^), PD1-expressing T (CD45^+^ CD3^+^ CD19^-^ PD1^+^), CD8 T (CD45^+^ CD3^+^ CD19^-^ CD8a^+^ CD4^-^), PD1-expressing CD8 T (CD45^+^ CD3^+^ CD19^-^ CD8a^+^ CD4^-^ PD1^+^), CD4 T (CD45^+^ CD3^+^ CD19^-^ CD8a^-^ CD4^+^), Treg (CD45^+^ CD3^+^ CD19^-^ CD8a^-^ CD4^+^ CD25^+^ FoxP3^+^), PD1-expressing CD4 T (CD45^+^ CD3^+^ CD19^-^ CD8a^-^ CD4^+^ PD1^+^), NKT (CD45^+^ CD3^+^ NK1.1^+^) and NK (CD45^+^ CD3^-^ NK1.1^+^) cells.


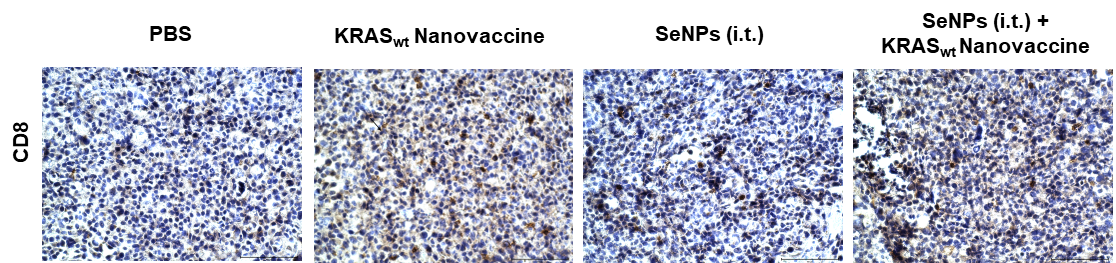


**Figure S11.** Representative images of EO771 tumors histology sections at day 20 with immunohistochemical staining for CD8 (brown). Cell nucleus stained in blue. Scale bars represent 100 µm.


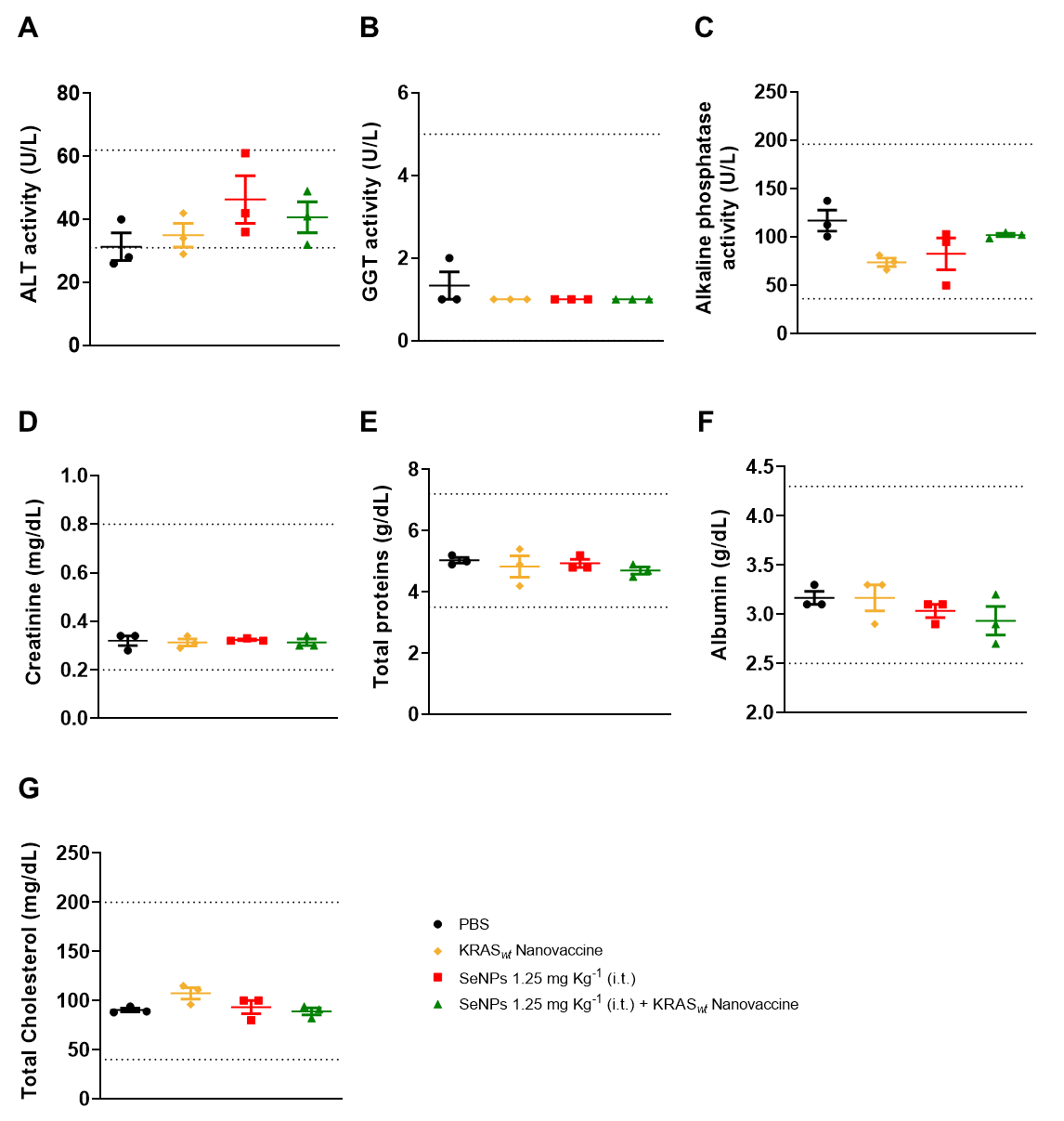


**Figure S12. Divalent combination of SeNPs intratumorally administrated with the KRAS*_wt_* nanovaccine induced a superior antitumor effect against EO771 luminal B breast cancer with no acute organ toxicity.** (**A to G**) Biochemical analysis of alanine aminotransferase (ALT) (**A**), gama glutamil transferase (GGT) (**B**), alkaline phosphatase (**C**) activities, as well as creatinine (**D**), total protein (**E**), albumin (**F**) and total cholesterol (**G**) levels in blood from animals of PBS, KRAS*_wt_* Nanovaccine, SeNPs 1.25 mg Kg^-1^ (i.t.), and SeNPs 1.25 mg Kg^-1^ (i.t.) + KRAS*_wt_* Nanovaccine groups. Data are presented as mean ± s.e.m of EO771-bearing mice (*n =* 3 animals). Statistical significance (not significant) was analyzed by one-way analysis of variance (ANOVA) followed by Tukey multiple comparisons post-hoc test).


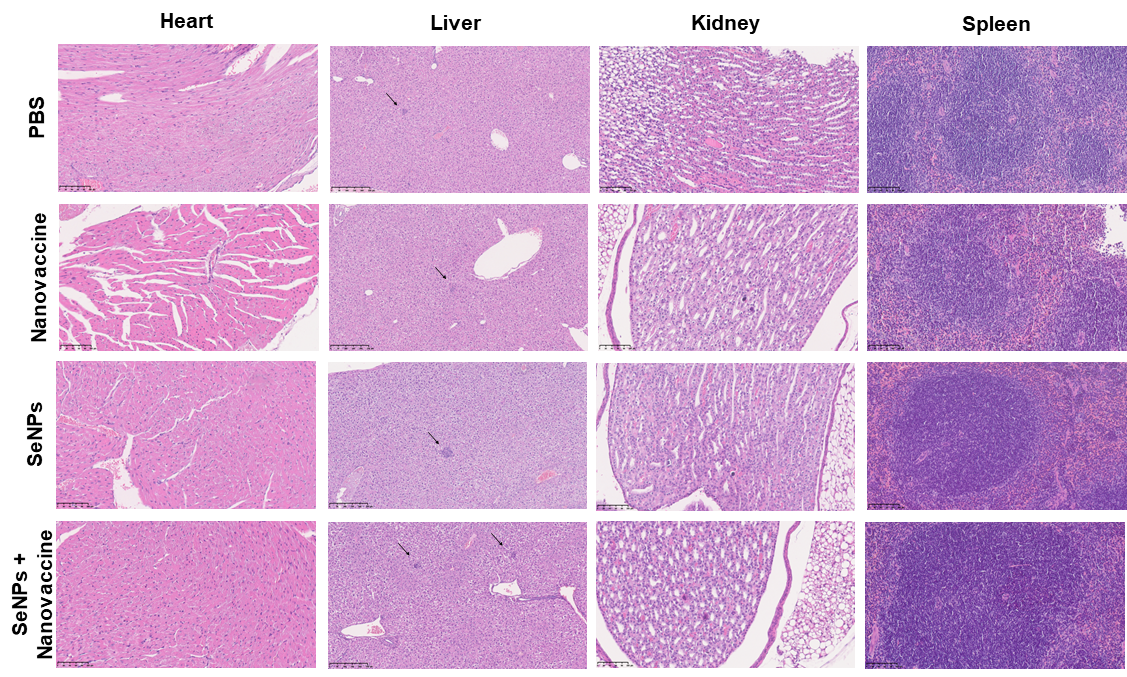


**Figure S13. Divalent combination of SeNPs intratumorally administrated with the KRAS*_wt_* nanovaccine induced a superior antitumor effect against EO771 luminal B breast cancer with no acute organ toxicity.** Representative histopathology images of hematoxylin and eosin staining of organs (heart (20X), liver (10X), kidney (20X), and spleen (20X)) recovered from animals treated with PBS, KRAS*_wt_* Nanovaccine, SeNPs 1.25 mg Kg^-1^ (i.t.), and SeNPs 1.25 mg Kg^-1^ (i.t.) + KRAS*_wt_* Nanovaccine. No significant alterations (within normal limits) were observed in the heart and spleen. Minimal renal mineralization with no clinical consequence was observed. Multifocal small foci of inflammatory cell infiltration (mononuclear microgranulomas) were found in the liver of animals from all groups.
